# Sex distorter male drive for resistance-resilient population control of the human malaria vector *Anopheles* *gambiae*

**DOI:** 10.1038/s41467-026-71627-1

**Published:** 2026-04-11

**Authors:** Silvia Grilli, Oksana Vertsimakha, Louise Marston, Irati Aramburu Gonzalez, Austin Burt, Andrea Crisanti, Federica Bernardini

**Affiliations:** Department of Life Sciences, Imperial College London, London, UK

**Keywords:** Biotechnology, Molecular engineering, CRISPR-Cas systems

## Abstract

Progress in malaria control has plateaued, prompting the exploration of additional tools. Here, we characterise two germline-specific promoters, *spo11* and *vasa1*, in the malaria vector *Anopheles gambiae*. These promoters display distinct temporal and spatial expression patterns, making them well-suited for potential applications in CRISPR-based gene drives and sex ratio distortion systems. Leveraging these unique promoter features, we developed a Sex Distorter Male Drive (SDMD) technology that generates a highly male-biased progeny while spreading through super-Mendelian inheritance. This approach greatly simplifies previous genetic construct designs, potentially improving genetic stability and resilience against the development of target site resistance, a major challenge for the efficacy of genetic strategies. Our findings position SDMD as a promising and potentially resistance-resilient tool for the population suppression of *Anopheles* mosquitoes in malaria-endemic regions.

## Abstract (web summary)

Malaria control has plateaued, driving the search for new mosquito control tools. Here, authors identify germline promoters in *Anopheles* *gambiae* that allow simplified resistance-resilient gene drive systems able to spread efficiently and produce strongly male-biased offspring.

## Introduction

Vector-borne diseases continue to pose significant threats to public health globally, claiming hundreds of thousands of lives every year, with malaria alone accounting for about 600,000 deaths in 2023^1^. Although control methods based on insecticides, insecticide-treated nets (ITNs) and anti-malarial drugs have helped to reduce this burden over the past decades, the decline in malaria incidence has recently stalled^1,2^.

Effective malaria control programmes often require an integrated approach that combines multiple strategies. Genetic tools, boosted by the revolutionary CRISPR-Cas9 system, are increasingly gaining the potential of becoming complementary vector control interventions. These strategies can be aimed at suppressing or replacing a target mosquito population, impacting its reproductive or pathogen-transmitting capabilities, respectively.

Based on their invasive dynamics and persistence, these technologies can further be classified into self-limiting or self-sustaining. Self-limiting technologies are characterised by impacts that are expected to be geographically restricted, requiring continuous inundative releases to achieve the desired effect. Conversely, self-sustaining strategies are anticipated to spread widely and persist in the target population following only small inoculative releases^3–7^.

Within self-sustaining strategies, significant progress has been made in developing CRISPR-Cas9-based gene drive technologies, which, in laboratory settings, have demonstrated potential for controlling mosquito populations^3–6^.

In the context of population suppression, homing-based gene drives are typically designed to disrupt genes that affect the fertility of female mosquitoes. However, this approach imposes a strong evolutionary pressure that can lead to the emergence and selection of functionally resistant alleles. This may eventually prevent the gene drive from spreading, hampering its long-term effectiveness^8,9^. To minimise the occurrence of resistance, highly conserved target sites must be selected as mutations occurring at these sites are likely to be non-functional and not favoured by natural selection^8,10,11^. An example of a target site for population suppression gene drives is the female-specific isoform of *doublesex* (*dsx*), a gene that plays a crucial role in regulating sexual dimorphism in *Anopheles*. Disruption of a highly conserved sequence at the boundary of intron 4 and exon 5 resulted in a female-specific recessive sterile phenotype with homozygous female mosquitoes exhibiting intersex features and unable to bite^11^. Such a phenotype was exploited to develop an efficient population suppression gene drive at this locus, for which the development of resistance was not detected in population invasion experiments^11,12^.

An alternative strategy for population suppression involves biasing the sex ratio of the mosquitoes’ offspring toward males^7^. This can be achieved by selectively shredding the X chromosome during male meiosis, ensuring that only Y-bearing sperm fertilise eggs^7,13–16^. In *Anopheles* mosquitoes, autosomal X-shredders have been engineered to target the 28S ribosomal DNA cluster, exclusively found on the X chromosome, using either the *I-PpoI*^13,17,18^ or Cas9^14^ endonucleases. These approaches have successfully led to the collapse of caged mosquito populations in laboratory experiments^14,17^. Notably, the development of resistance against the nucleases’ cleavage activity is mitigated by the highly repetitive nature of the X-linked target sites. If engineered on the Y chromosome, such a sex distorter system would result in a powerful and self-sustaining population suppression strategy known as Y-drive^7^. However, while it has been possible to express transgenes from the Y chromosome of *A. gambiae* at early stages of spermatogenesis^19,20^, meiotic sex chromosome inactivation (MSCI)^21,22^ has impeded Y-drive development^13,23^ due to the transcriptional silencing of the sex chromosomes during meiotic stages of male gametogenesis.

A strategy to achieve super-Mendelian inheritance of a sex distorter system has recently been developed in *A. gambiae* by combining the *I-PpoI* nuclease with a CRISPR-based gene drive targeting the female isoform of *dsx*^24^. This system, referred to as sex distorter gene drive (SDGD), employs the regulatory sequences of *zero population growth* (*zpg*) to drive Cas9 expression and enable homing of the construct in both sexes. In addition, the male-specific *ß2-tubulin* (*ß2-tub*) promoter drives expression of the *I-PpoI* during the meiotic stages of spermatogenesis, leading to X chromosome shredding. As a result, heterozygous SDGD males generated super-Mendelian inheritance of the construct and a male-biased progeny, while the rare female escapees were fertile, further contributing to the spread of the drive in laboratory mosquito populations^24^.

**Fig. 1:**
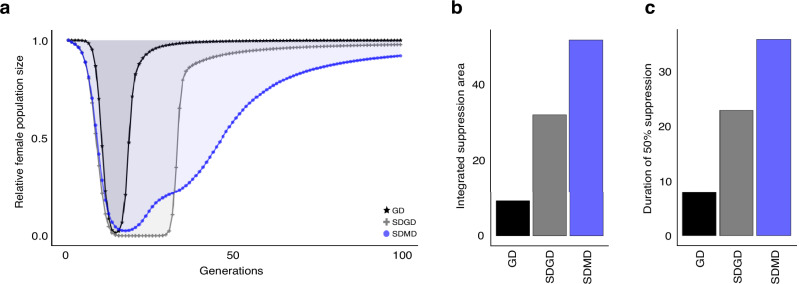
Predicted effect of including sex ratio distortion on the evolution of resistance to a population suppression gene drive. **a** Relative number of fertile females across generations following the release of gene drive (GD, black), SDGD (grey) and sex-distorting male drive (SDMD, purple) at 10% of the initial population size (*N* = 10^15^). For all the strategies, the relative number of females initially declines due to the gene drive, and then recovers at different rates due to the evolution of resistance. **b** Mean integrated suppression area (i.e. the area above the corresponding curve in **a**) and **c** the average number of generations with >50% suppression of the females in the population. Incorporating sex distortion leads to an increase in the duration of protection and the integrated suppression area values, and the increase is even greater for the SDMD strategy, where drive-bearing females are sterile. Modelling results with generalised fitness parameters (Supplementary Tables 5 and 6), cleavage rate *c* = 0.95, HNEJ rates *j* = 0.035, and fraction of functional resistance *b* = 0.005. Sex distortion for SDGD and SDMD strategies is 95%. The outputs were averaged over 100 simulations.

Compared to a gene drive targeting the *dsx* gene, the sex distortion component in the SDGD leads to a faster reduction of the number of females in the population, accelerating the potential impact on disease transmission dynamics and reducing the opportunity for functional target site resistance to evolve before elimination^24^. Interestingly, the development of resistance against an SDGD could be further reduced by ensuring that the rare escapee females are sterile. In this scenario, since the individuals undergoing positive selection for the emergence of functional resistance, i.e. the females, would be unable to pass any resistant allele on to the next generation, the population suppression effect would be longer-lasting and more effective (Fig. 1).

A simplified SDGD construct could be envisioned using a Cas9 nuclease under the control of a single germline promoter to simultaneously induce homing and X-shredding by employing two gRNAs, one targeting an autosomal female-fertility gene and the other the X-linked 28S rDNA cluster. However, this approach presents the challenge of achieving two biological processes in the mosquito’s germline that should occur at different optimal timeframes: homing is favoured early during spermatogenesis when homology-directed repair (HDR) is preferred over non-homologous end joining (NHEJ), while the ideal expression window for X-shredding is during meiosis, as disrupting the X chromosome at the early stages of gametogenesis would likely result in meiotic arrest and sterility^13,14,25^.

In this study, we conducted a thorough investigation of the regulatory sequences of two germline genes: *spo11* (AGAP010898), a highly conserved topoisomerase catalysing the formation of double-strand breaks during early stages of meiosis leading to recombination^26–32^, and *vasa* (AGAP008578), a gene required in early development and crucial in establishing the germline lineage^33–35^.

The regulatory sequences of these genes were selected based on their transcriptional profile across mosquito gametogenesis. In *Anopheles*, *spo11* is expressed in the germline of both sexes: in the testes, transcripts accumulate predominantly in the region corresponding to early meiotic stages of spermatogenesis, while being absent from the apical tip where the stem cell niche resides; in females, *spo11* expression is induced in the ovaries following a blood meal^36^.

In a previous study, the *vasa1* promoter was characterised as male-specific, capable of driving gene expression during spermatogenesis at early meiotic stages^35^.

To our knowledge, neither the *spo11* nor the *vasa1* promoters have been further explored for vector control applications in *Anopheles*. As both promoters are expressed later than those previously used to efficiently induce homing, such as *vasa2*, *zpg* or *nanos* (*nos*), but earlier than *ß2-tub*, which has been used for developing X-shredding sex distorters, we investigated the potential of *spo11* and *vasa1* both for homing and sex distortion strategies.

In this study, we report two promoters, *spo11* and *vasa1*, that enable both homing and sex distortion. Leveraging this unique dual functionality, we developed a highly efficient genetic system targeting the *dsx* locus, which we term the sex distorter male drive (SDMD). Heterozygous SDMD males exhibit super-Mendelian inheritance alongside a strong male-biased progeny, while the rare females, hereafter referred to as “female escapees”, are sterile. This approach simplifies previous SDGD designs and, by ensuring the sterility of the rare female escapees, further reduces the likelihood of selection for resistance. This work significantly expands the toolbox for the development of additional vector control technologies and provides valuable insights into the biology of mosquito gametogenesis.

## Results

### Development of transgenic strains to test the promoters of *spo11* and *vasa*

**Fig. 2:**
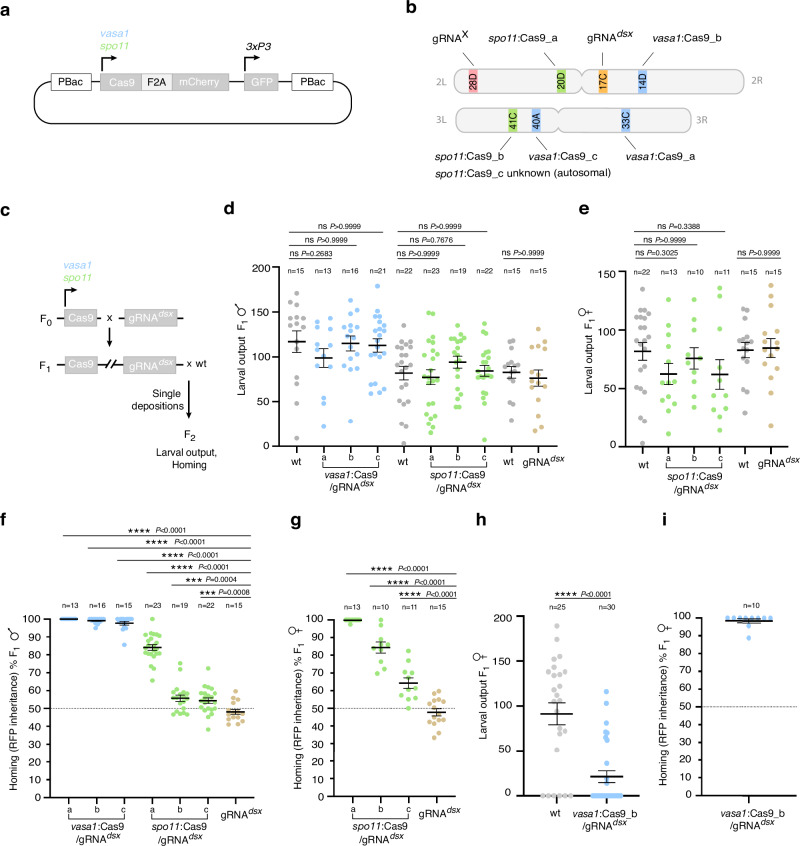
*vasa1* and *spo11*:Cas9 constructs and homing assessment. **a** Schematic representation of the *vasa1*:Cas9 and *spo11*:Cas9 constructs. **b** Schematic representation of the transgene’s genomic location. Integration site of *spo11*:Cas9_c is unknown, but the inheritance pattern of the transgene suggests autosomal insertion. **c** Schematic representation of the genetic crosses used to evaluate transgene inheritance. The transgenic cassette in the gRNA^*dsx*^ strain, inserted in the *dsx* gene, includes a *3xP3*:RFP fluorescent marker used to score RFP inheritance. **d**–**i** The progenies of F_1_ trans-heterozygotes *vasa1*:Cas9/gRNA^*dsx*^ (blue) and *spo11*:Cas9/gRNA^*dsx*^ (green) males (**d**, **f**) and females (**e**, **g**–**i**) were counted individually (**d**, **e**, **h**) and screened for RFP (**f**, **g**, **i**) to assess fecundity and inheritance rates, respectively. Wild type (grey) and gRNA^*dsx*^ (beige) males and females were used as controls. Dots represent the progeny of a single mosquito; the number of progenies analysed is indicated on top of each data group (sample size, *n*). Thick horizontal lines with error bars indicate the arithmetic mean and standard error of the mean (s.e.m.), respectively. Statistical analysis of larval output was performed using the Kruskal–Wallis test followed by two-sided Dunn’s multiple comparison test with wild type control (**d**, **e**) or two-sided Mann–Whitney test (**h**). Statistical analysis of RFP inheritance was evaluated by comparing the total number of RFP positive and negative individuals in the experimental group *versus* their respective wild-type controls (Fisher’s exact test, two-sided). *P* values are indicated in the figure above each data group. Independent experiments were performed for each group (*vasa1*:Cas9/gRNA^*dsx*^, *spo11*:Cas9/gRNA^*dsx*^ and gRNA^*dsx*^), each including a wild-type control, which was used for the corresponding statistical analysis. Differences among wild-type groups reflect natural biological variation between independent experiments. Source data are provided as a Source Data file. Graphs and statistical analysis were generated using GraphPad Prism (v10.6.0).

To investigate the regulatory regions of *spo11* and *vasa* as gene-editing tools in *Anopheles*, we designed genetic constructs to express the Cas9 endonuclease under these sequences (Fig. 2a). For the *vasa* gene, we used the previously characterised *vasa1* promoter, which encompasses about 1.8 kb upstream of the transcription start site and includes the first untranslated exon^35^. To isolate the putative promoter of *spo11*, we amplified approximately 2.8 kb upstream of the translation start site annotated in the AgamP4 reference genome, encompassing the entire 5’UTR (1036 bp) and the flanking 1842 nucleotides. In both constructs, the promoter was engineered immediately upstream of the coding sequence of the Cas9 endonuclease, which was linked to a mCherry fluorescent marker through an F2A self-cleaving peptide, aiming at visualising the expression pattern of the nuclease under the control of the same regulatory sequences. Approximately 1 kb was amplified downstream of the stop codon of each gene to serve as terminators and engineered at the 3’ end of the Cas9-F2A-mCherry cassette. Each construct also included a *3xP3*:GFP fluorescent marker to identify transgenic individuals. Three independent *spo11*:Cas9 and *vasa1*:Cas9 transgenic *A. gambiae* strains were generated through PiggyBac semi-random integration, each harbouring a single autosomal integration of the construct, identified through iPCR (Fig. 2b and Supplementary Table 1). A fertility assay conducted on both heterozygous males and females of each strain mated to wild type mosquitoes revealed no fitness costs associated with the insertion of the transgene in any of the strains (Supplementary Fig. 1). Notably, we did not detect any mCherry fluorescence signal in the reproductive organs of these individuals, likely due to either weak activity of these promoters and/or the influence of the ribosome stuttering 2A sequence on the expression of the linked peptide chain.

### The regulatory sequences of *spo11* and *vasa* facilitate homing in the germline

To investigate the ability of the *spo11* and *vasa1* promoters to effectively drive Cas9 expression at a timeframe suitable to induce homing, we analysed the inheritance of a transgene-linked RFP in the progeny of *spo11*- or *vasa1*:Cas9/gRNA^*dsx*^ trans-heterozygous individuals (Fig. 2c). The gRNA^*dsx*^ strain was previously generated in our laboratory, and it harbours a U6:gRNA cassette linked to a *3xP3*:RFP marker inserted at the target *locus*, i.e. disrupting the female-specific intron 4 - exon 5 boundary of *dsx*^11^ and leading to an intersex phenotype in female homozygous individuals.

For the *spo11* promoter, assays were conducted in both sexes, while only males were initially assessed for *vasa1*, because of the male-specific expression reported in literature^35^.

No significant differences were identified in the larval output of trans-heterozygous *spo11*:Cas9/gRNA^*dsx*^ males and females, nor in trans-heterozygous *vasa1*:Cas9/gRNA^*dsx*^ males (Fig. 2d, e), suggesting the absence of unintended fertility impairment. Notably, both the *vasa1* and *spo11* promoters exhibited the ability to express the Cas9 endonuclease at suitable timeframes to drive the inheritance of the gRNA^*dsx*^ at super-Mendelian rates (Fig. 2f, g). However, while all the *vasa1*:Cas9 strains induced high homing rates (≥97.7% ± 1.0 s.e.m.), higher variability (ranging from 55.8% to 99.8%) was observed across the *spo11*:Cas9 strains, presumably reflective of positional effects (Fig. 2f, g). The *spo11*:Cas9_a strain outperformed the other two, exhibiting strong super-Mendelian inheritance both in males (83.0% ± 1.5 s.e.m) and females (99.8% ± 0.2 s.e.m), irrespective of paternal (Fig. 2f, g) or maternal (Supplementary Fig. 2) Cas9 inheritance in the parental (F_0_) cross.

### *Vasa1* expression detected in female gametogenesis

While generating trans-heterozygotes *vasa1*:Cas9/gRNA^*dsx*^ individuals, we noticed that a small fraction of females, representing <1% of each strain, showed a mosaic phenotype with intermediate sex features at the pupal genital lobe, hereafter referred to as ‘sexual mosaicism’ (Supplementary Table 2). Despite *vasa1* being previously described as male-specific^35^, this observation suggested that this promoter might also be active in females. Such a hypothesis was further supported by the presence of mutagenesis at the *dsx* target site in the trans-heterozygotes *vasa1*:Cas9/gRNA^*dsx*^ females analysed (Supplementary Fig. 3). To elucidate this phenotype and explore whether *vasa1* could also drive Cas9 expression in the female germline, we assessed the fecundity and homing efficiencies of the *vasa1*:Cas9_b/gRNA^*dsx*^ trans-heterozygous females. Notably, only one-third of the individuals tested (*n* = 10/30) generated viable progeny, and their larval output was also significantly reduced (63.7 ± 10.5 s.e.m.) compared to wild types (120.1 ± 8.4 s.e.m), indicative of a severe fitness cost (*t*-test, ^***^P < 0.001) (Fig. 2h). Interestingly, in such progenies, a high number of individuals (98.5% ± 1.1 s.e.m) inherited the transgene, reflecting super-Mendelian inheritance of the gRNA^*dsx*^ construct (Fig. 2i).

These observations demonstrated that the *vasa1* promoter is also expressed in the female germline and can be used to induce homing efficiently in *Anopheles*. The observed sexual mosaicism and fitness cost in the trans-heterozygotes *vasa1*:Cas9/gRNA^*dsx*^ females possibly reflect the somatic leakiness of this promoter, similar to previous observations with *vasa2* and *zpg* promoters^9–11,37^.

### The regulatory sequences of *spo11* and *vasa1* lead to high male bias

**Fig. 3:**
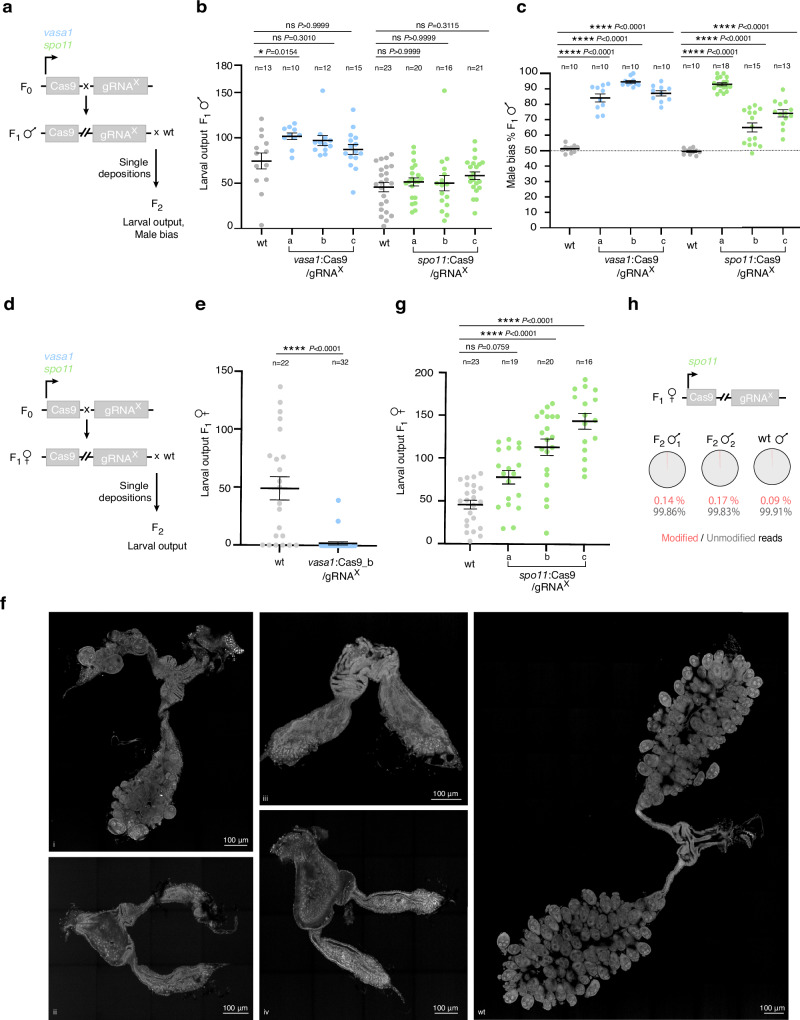
X-shredding assessment using *vasa1* and *spo11* promoters. **a** Schematics of the genetic crosses to evaluate fecundity (**b**) and sex ratio distortion (**c**) of *vasa1*:Cas9/gRNA^X^ (blue) and *spo11*:Cas9/gRNA^X^ (green) males in comparison to wild type (grey). **d** Schematics of the genetic crosses used to evaluate X-shredding in females. **e** Larval output and **f** DAPI staining of reproductive organs (*n* = 4) of *vasa1*:Cas9/gRNA^X^ trans-heterozygous females and wild type control (scalebar = 100 μm). Defects in reproductive organs were consistently observed in a total of 15 independent dissections. **g** Larval output of *spo11*:Cas9/gRNA^X^ trans-heterozygous females. Each dot represents the progeny of a single female mosquito, and the number of progenies analysed is indicated on top of each data group (sample size, *n*). Thick horizontal lines with error bars indicate the arithmetic mean and s.e.m., respectively. Statistical analysis of larval output was conducted using the Kruskal–Wallis test followed by two-sided Dunn’s multiple comparison test with the wild type control (**b**, **g**) or two-sided Mann–Whitney test (**e**). Male bias statistical analysis was performed by comparing the total number of males and females in the experimental group *versus* their respective wild-type controls (Fisher’s exact test, two-sided). *P* values are indicated in the figure above each data group. Independent experiments were performed for each group (*spo11*:Cas9, *vasa1*:Cas9 and gRNA^*dsx*^ strains), each including an internal wild-type control, which was used for the corresponding statistical analysis. Differences among wild-type groups reflect natural biological variation between independent experiments. Source data are provided as a Source Data file. **h** Proportion of modified reads at the rDNA target site of the male progeny of *spo11*:Cas9/gRNA^X^ trans-heterozygous females, analysed through pooled amplicon sequencing (two biological replicates, *n* = 30; wt *n* = 30). Graphs and statistical analysis were generated using GraphPad Prism (v10.6.0).

To evaluate the potential of generating male-biased progeny using either *vasa1* or *spo11* regulatory sequences, we mated each *vasa1- or spo11*:Cas9 strain with the gRNA^X^ strain previously generated in our laboratory. In this gRNA^X^ strain, the transgene has been inserted on chromosome 2L and includes the U6:gRNA cassette targeting the X-linked 28S rDNA repeats, previously used to develop a CRISPR-based sex distorter in *A. gambiae*^14^. Trans-heterozygous *vasa1*- or *spo11*:Cas9/gRNA^X^ males were outcrossed to wild types (Fig. 3a), and progeny of individual females were analysed to assess fertility (Fig. 3b) and sex ratio (Fig. 3c). Notably, in these crosses, we did not observe any reduction in fertility compared to wild type controls (Fig. 3b), while the sex ratio in the progeny was significantly skewed toward males (Fig. 3c). Specifically, all the three *vasa1*:Cas9/gRNA^X^ genetic crosses resulted in high average male bias (ranging from 84.2% ± 2.6 s.e.m to 94.6% ± 0.9 s.e.m.). In contrast, the X-shredding efficiency of *spo11*:Cas9 showed more variability across the three strains, recording average male bias values ranging from 64.9% ± 2.9 s.e.m to 93.0% ± 1.0 s.e.m, possibly due to positional effects (Fig. 3c). These results demonstrated the potential of *vasa1* and *spo11* promoters to bias the transmission of gametes in *A. gambiae* pre-zygotically, providing additional tools in the context of sex distorter techniques.

### Evaluating the impact of X-shredding in female gametogenesis

Thus far, the only known promoter for engineering X-shredders in insects is the *ß2-tub*^13–16,18,24^, which is active exclusively in the germline of male individuals.

In this study, we have demonstrated that pre-zygotic bias of Y-bearing gametes in *A. gambiae* can also be induced using *vasa1* and *spo11* promoters. Since these two promoters are active in both sexes, we next sought to address the effect of deliberately inducing X-shredding in females (Fig. 3d), which could provide insights into mosquito oogenesis and explore the potential of employing such promoters for vector control strategies.

Because females only produce X-bearing gametes, we hypothesised that shredding of both X chromosomes causes sterility. As expected, trans-heterozygous *vasa1*:Cas9/gRNA^X^ females revealed a severe fitness cost, with only about 6% of the individuals tested (*n* = 2/32) being able to generate viable progeny, although exhibiting a reduced larval output (20.3 ± 11.0 s.e.m) when compared to wild types (67.4 ± 10.4 s.e.m) (Fig. 3e). The high fitness cost observed is correlated to a severe underdevelopment of the reproductive organs of trans-heterozygous *vasa1*:Cas9_b/gRNA^X^ females, which exhibited atrophic ovaries whereby ovarioles were partially or totally absent (Fig. 3f).

Unexpectedly, the trans-heterozygous *spo11*:Cas9/gRNA^X^ females were fertile, generating high counts of viable progeny (*spo11*:Cas9_a/gRNA^X^ 77.8 ± 7.8 s.e.m., *spo11*:Cas9_b/gRNA^X^ 113.2 ± 9.6 s.e.m., *spo11*:Cas9_c/gRNA^X^ 143.9 ± 9.2 s.e.m., wild type 45.8 ± 5.2 s.e.m., Kruskal–Wallis test ^****^P < 0.0001) (Fig. 3g).

Having previously demonstrated that the *spo11* promoter is active in females, further analyses were performed to investigate this phenotype. First, we assessed whether the activity of the Cas9 nuclease mutagenized the X chromosome by performing amplicon sequencing at the rDNA target site on the male offspring of *spo11*:Cas9/gRNA^X^ trans-heterozygous females. This revealed some but rare indels at the rDNA target site, which only accounted for <0.2% modification of the total reads (Fig. 3h), suggesting either minimal damage to the rDNA repeats or prompt DNA repair, for instance, through either HDR or intramolecular recombination using the rDNA repeats present on the same chromosome as template^38^.

To further investigate this phenotype, we conducted a fertility assay on the offspring of the *spo11*:Cas9/gRNA^X^ trans-heterozygous females to assess fitness costs resulting from a potentially damaged X chromosome inherited maternally. Such a study revealed neither a significant fitness cost in these individuals nor sex bias in their progeny (Supplementary Fig. 4).

These observations highlight complex dynamics when the X chromosome is targeted in the female gametogenesis, which could be correlated to different dynamics of X chromosome accessibility or repair of highly repetitive sequences^38^.

### Building an SDMD in *dsx* using the *vasa1* promoter

Aiming at optimising the previously developed SDGD^24^, we leveraged the characteristics of *spo11* and *vasa1* promoters to investigate the feasibility of employing a single promoter to bias Mendelian inheritance and sex ratio simultaneously. As mathematical modelling predicted that, in an SDGD system, the sterility of the rare female escapees would further mitigate the emergence of resistance (Fig. 1), we opted for engineering the Cas9 endonuclease under the regulatory sequences of *vasa1*. This is because, based on our observation in the *vasa1*:Cas9/gRNA^X^ trans-heterozygous individuals, X-shredding during female gametogenesis would be expected to cause sterility when *vasa1*, but not *spo11* promoter, is employed (Fig. 3d–g). Furthermore, the *vasa1* promoter showed somatic leakiness, which, combined with the gRNA^*dsx*^, resulted in sexual mosaicism and a significant fitness cost in female mosquitoes (Figs. 2h, i and S3). Conversely, this phenotype was not observed with the *spo11* promoter, for which the fitness of trans-heterozygous *spo11*:Cas9/gRNA^*dsx*^ females did not statistically differ from the wild type control (Figs. 2e and S2). Additionally, the *vasa1* promoter showed less susceptibility to positional effects than *spo11* (Figs. 2f, g and 3c), therefore being more promising for inducing high levels of homing and male bias.

**Fig. 4:**
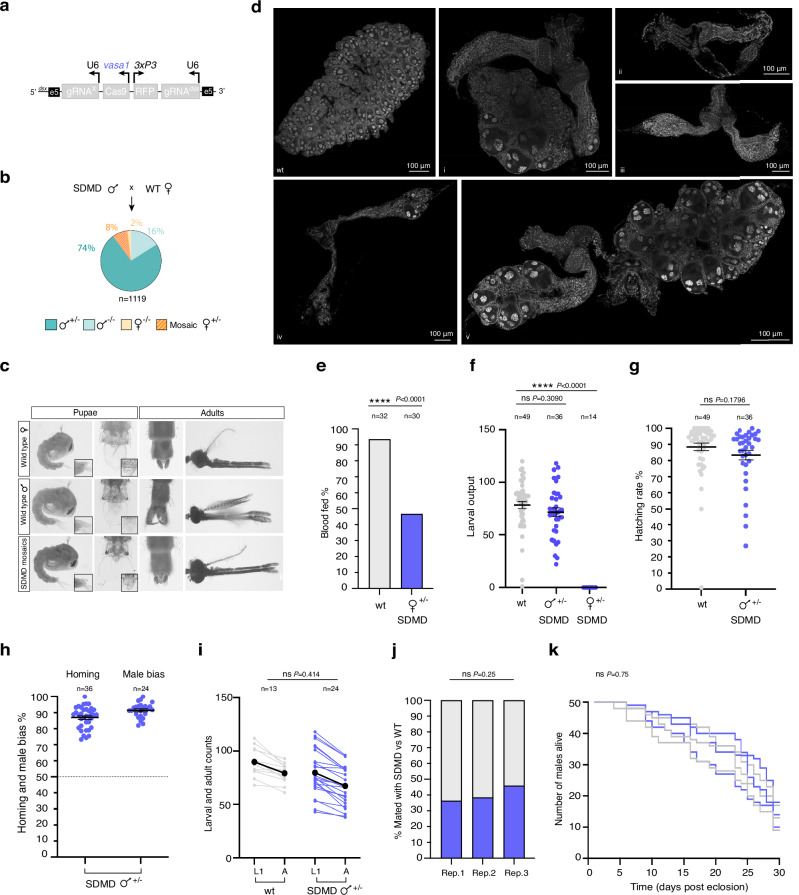
SDMD in *dsx.* **a** Schematic representation of the SDMD construct. **b** Proportion of the genotypes composing the progeny of SDMD males (♂/♀ ^+/^^−^ = SDMD heterozygotes; ♂/♀^−/−^ = siblings not inheriting the transgene). **c** Sexual mosaicism of SDMD females. The phenotype shown reflects observations made in 20 individuals. **d** DAPI staining of reproductive organs (*n* = 5) of SDMD and wild type females (scalebar = 100 μm). Defects in reproductive organs were consistently observed in a total of 20 independent dissections. **e** Percentage of blood-fed SDMD (purple) (*n* = 14/30) and wild type (*n* = 29/32) (grey) females (Fisher’s exact test, two-sided). **f** Larval output, **g** hatching rate, **h** homing and male bias of the progeny of SDMD (purple) and wild type (grey) males. Each dot represents a single progeny (sample size, *n*). Horizontal lines with error bars indicate the arithmetic mean and s.e.m., respectively. Statistical analysis of larval output was performed using the Kruskal–Wallis test followed by two-sided Dunn’s multiple comparison test. Statistical analysis of hatching rate was performed by comparing the number of eggs and larvae in the experimental group *versus* the wild type control (Fisher’s exact test, two-sided). **i** Larval to adult survival was measured by comparing the counts of first instar larvae and adults from each progeny (Fisher’s exact test, two-sided). **j** Percentage of wild type females (*n* = 50) mated with either SDMD (*n* = 50) or wild type (*n* = 50) males in the mating competitiveness assay across three replicates (Wilcoxon matched-pairs signed-rank test, two-sided). **k** Thirty-days survival of SDMD adult males (*n* = 50) and wild types (*n* = 50) across three replicates (Wilcoxon matched-pairs signed-rank test, two-sided). *P* values are indicated in the figure above each data group. Source data are provided as a Source Data file. Graphs and statistical analysis were generated using GraphPad Prism (v10.6.0).

Based on this, we engineered an SDMD technology at the previously characterised intron 4-exon 5 boundary of *dsx*^11,24^, harbouring the Cas9 endonuclease under the *vasa1* regulatory sequences and two gRNAs, each independently expressed under a U6 Pol III transcription unit. To home the construct at the corresponding *dsx* allele, we employed the gRNA^*dsx*^, while the gRNA^X^ was included to induce X-shredding by targeting the X-linked 28S rDNA repeats (Fig. 4a).

A preliminary investigation of the phenotype of the SDMD strain was conducted by crossing in bulk males heterozygous for the transgenic construct to wild type females and analysing the genotypes in their progeny (Fig. 4b). Remarkably, we observed high male bias (90%) and super-Mendelian inheritance of the transgene (82%), suggesting that both the homing and the X-shredding reactions may have been induced. A small proportion of females was also generated, corresponding to about 10% of the total progeny. Among this cohort, those harbouring the transgene accounted for 8% of the total progeny. These individuals showed sexual mosaicism (Fig. 4c), underdeveloped ovaries (Fig. 4d), impairment in their ability to feed on blood (Fig. 4e) and full sterility (Fig. 4f). The remaining 2% of the female escapees did not inherit the transgene and were fertile (Supplementary Fig. 5).

To further characterise this strain, heterozygous SDMD males were crossed in bulk to wild-type females, which, after a blood meal, were allowed to lay eggs individually. In the progenies, we did not record any significant difference in larval output (Fig. 4f) or hatching rate (Fig. 4g) when compared to the wild type controls, suggesting that the SDMD construct did not impair male fertility. Notably, the heterozygous SDMD males efficiently combined homing and X-shredding, resulting in an average transgene inheritance rate of 86.9% ± 1.1 s.e.m, and a significant sex ratio distortion toward males (91.4% ± 0.9 s.e.m) (Fig. 4h). Importantly, the larval-to-adult survival rate of the progeny of SDMD heterozygous males did not statistically differ from that of wild types (Fig. 4i), suggesting that the sex bias originated pre-zygotically and was not caused by mortality of the female progeny.

To explore whether the non-transgenic progeny of SDMD heterozygous males did not inherit the transgene due to mutations at the *dsx* target site, which would prevent the homing reaction, we performed pooled amplicon sequencing at the gRNA^*dsx*^ target site. This analysis revealed a range of indels accounting in total for less than 6% of the reads, indicating some, but rare, end-joining events (Supplementary Fig. 6). Further investigation attributed the presence of these mutated alleles to Cas9 activity in the parental germline (Supplementary Fig. 7).

To further assess the fitness of the SDMD heterozygous males, we conducted a mating competitiveness assay. Three replicate cages were set up, each consisting of 50 SDMD males, 50 wild type males and 50 wild type females (1:1:1 ratio). Females were allowed to mate for 5 days, after which they were provided with a blood meal and individually separated into cups for oviposition the following day. Progeny were scored for the presence or absence of the transgene, indicating whether females had mated with an SDMD or wild-type male, respectively.

Overall, a lower proportion of females (36–45%) mated with SDMD males compared to wild type (55–64%). However, this difference was not statistically significant (Wilcoxon matched-pairs signed-rank test, *P* = 0.25) (Fig. 4j).

Male survival was monitored over 30 days following pupal eclosion by collecting dead individuals and analysing them for the presence of the transgene. No significant difference in survival was observed between SDMD and wild-type males in any replicate, indicating that the construct does not affect adult male longevity (Fig. 4k).

### Exploring the sexual mosaicism of the SDMD female escapees

**Fig. 5:**
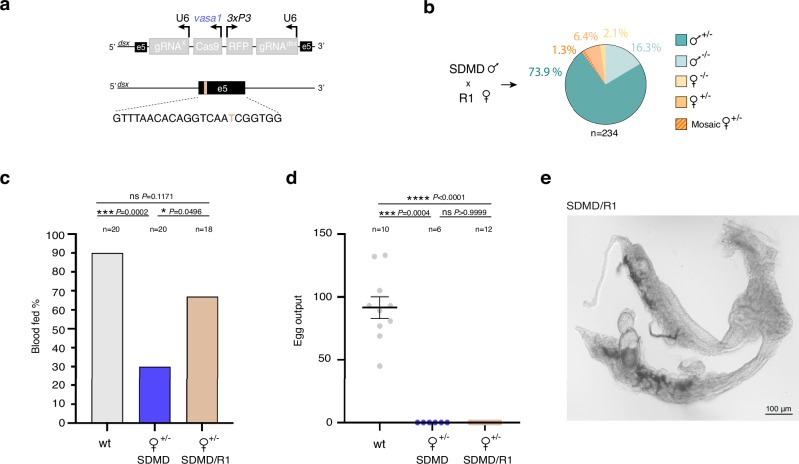
Phenotype of the SDMD female in combination with a functional resistant (R1) allele at the *dsx* target site. **a** Schematic representation of the *dsx* locus in SDMD/R1 trans-heterozygous individuals. The R1 allele consists of a G > T SNP (beige) at position 17 of the gRNA^*dsx*^, which confers full resistance against the gene drive^9^, preventing mutagenesis at the *dsx* locus, while X-shredding is unaffected. **b** Proportion of the genotypes composing the progeny of heterozygous SDMD males crossed in bulk to R1 females, showing the partial rescue of the sexual mosaic phenotype in SDMD/R1 females (♂/♀ ^+/^^−^ = SDMD heterozygotes; ♂/♀^−/−^ = siblings not inheriting the transgene) **c** Proportion of blood fed SDMD (*n* = 6/20) (purple) and SDMD/R1 (*n* = 12/18) (beige) females compared to wild type (*n* = 18/20) (grey) (Fisher’s exact test, two-sided). **d** Egg output of blood-fed SDMD and SDMD/R1 females. Dots represent the progeny of a single female mosquito. Thick horizontal lines with error bars indicate the arithmetic mean and s.e.m., respectively. Statistical analysis of egg output was performed using the Kruskal–Wallis test followed by two-sided Dunn’s multiple comparison test with the wild type control. **e** Reproductive organs of an SDMD/R1 female are showing atrophic ovaries. The defective phenotype was consistently observed in all 18 SDMD/R1 individuals analysed, and a representative image is shown. Scale bar = 100 μm. *P* values are indicated in the figure above each data group. Source data are provided as a Source Data file. Graphs and statistical analysis were generated using GraphPad Prism (v10.6.0).

The sterility of the SDMD female escapees is a key aspect of our technology compared to the previously developed SDGD^24^. Thus, the underlying causes of such a phenotype were investigated in more detail. Based on the significant fitness impairment previously observed in the trans-heterozygous *vasa1*:Cas9_b/gRNA^*dsx*^ and *vasa1*:Cas9_b/gRNA^X^, we hypothesised that the sterility in the SDMD female escapees could be due to both leaky expression of Cas9 in somatic tissues, leading to sexual mosaicism, and to X-shredding during oogenesis, resulting in ovary atrophy. To investigate this phenotype, we leveraged the availability of a previously developed *A. gambiae* strain, hereafter referred to as R1^*dsx*^, which carries a functional resistant allele at the *dsx* target site^9^ (G > T SNP at position 17 of the gRNA^*dsx*^) (Fig. 5a). This allele has been shown to both completely block the homing activity of a gene drive targeting the gRNA^*dsx*^ target site and rescue the fertility of heterozygous gene drive females, whose sexual mosaicism and impaired fertility were attributed to somatic leakiness and/or deposition of Cas9^9^.

A genetic cross was performed between heterozygous SDMD males and females homozygous for the R1^*dsx*^ allele, and the phenotype of the female escapees in their progeny was assessed (Fig. 5a, b). We observed a rescue of the sexual mosaicism in more than 80% of the trans-heterozygous SDMD/R1 females analysed (*n* = 15/18) (Fig. 5b). Such a phenotype rescue was also reflected by a significantly higher proportion of individuals able to take a blood meal in comparison to SDMD female escapees (Fig. 5c). Importantly, none of these females generated any viable progeny (Fig. 5d), and showed underdeveloped ovaries (Fig. 5e), indicating persistence of the sterile phenotype even in the presence of the R1^*dsx*^ allele, likely attributed to the activity of Cas9 at the X-linked rDNA locus.

In conclusion, the R1^*dsx*^ allele largely rescued the sexual mosaic phenotype of the SDMD female escapees. Nevertheless, the SDMD/R1^*dsx*^ trans-heterozygous females remained sterile, revealing that the gRNA^X^ is sufficient to cause sterility in the SDMD female escapees.

### Mathematical modelling predicts a strong effect of the SDMD on population suppression

**Fig. 6:**
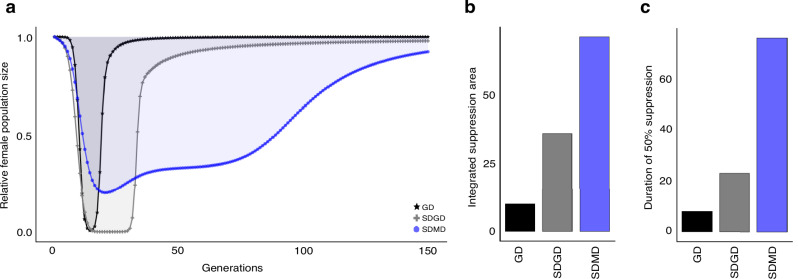
Modelling of the population suppression effect for specific GD, SDGD, and SDMD constructs targeting *dsx.* **a** Relative number of fertile females across generations following the release of gene drive (GD, black), SDGD (grey) and SDMD (purple) at 10% of the initial population size (*N* = 10^15^). The shaded area corresponds to the suppression due to each gene drive release. **b** Mean integrated values of the suppression area (i.e. the area above the corresponding curve in (**a**)) for 150 generations. **c** Average number of generations with 50% female population suppression. Parameters were set according to the empirical values for specific constructs (Supplementary Tables 5 and 7). The outputs were averaged over 100 simulations.

Next, we used mathematical modelling to investigate the effect of the SDMD technology on a target mosquito population. The dynamics following the release of SDMD males at 10% of the initial population size (*N* = 10^15^) were predicted using the empirical parameters observed in the *dsx* locus in this study and compared to those of the previously developed SDGD^24^ and GD^9^. Overall, the SDMD showed greater resilience against both non-functional and functional resistance, resulting in a more lasting effect on population suppression, as the population recovery is slower compared to the SDGD and GD (Fig. 6a–c). The simulations demonstrated the advantages of the SDMD for long-term population suppression, maintaining a 50% reduction of the female population for approximately 75 generations. This significantly outperformed the SDGD and the GD, which sustained similar suppression levels for less than 25 and 10 generations, respectively (Fig. 6c).

In conclusion, by mediating the sterility in female escapees, the SDMD reduces the proportion of individuals experiencing positive selection of functional resistance, enhancing the duration of the population suppression effect compared to GD and SDGD technologies. This highlights its potential as a promising tool for genetic vector control.

## Discussion

A major challenge undermining the efficacy of homing-based vector control strategies is the emergence of resistance at the target site, as such alleles prevent the homing reaction and ultimately block the spread of the drive^8,9,37^.

Several strategies have been proposed to mitigate resistance. Targeting functionally constrained sequences can limit the accumulation of functional and nonfunctional resistant alleles, as demonstrated in laboratory studies^11,24,37^. Additionally, using multiplexed gene drives is expected to significantly decrease the probability of resistance, especially when targeting non-overlapping sites that exhibit strong sequence constraints^9,11,39^. Importantly, nuclease activity must be restricted to a specific timeframe in the germline when homologous-directed repair (HDR) can be favoured over the error-prone NHEJ pathway. This is crucial to enhance homing efficiency and minimise somatic leakiness and deposition of the CRISPR-Cas9 complex into the embryo, which can result in unintended fitness costs and formation of resistant alleles.

Homing-based gene drives in *Anopheles* have been developed using the *cis*-regulatory elements of genes expressed early in male and female gametogenesis, such as *vasa2*^10,40,41^*, zpg*^11,37^ and *nanos (nos)*^37^. However, maternal deposition of the nuclease (*vasa2, nos*) and somatic leakiness (*vasa2, zpg*) pose challenges to the effectiveness of such molecular tools^8,10,11,37^.

This study thoroughly investigated two germline promoters, *spo11* and *vasa1*, to assess their potential to minimise the nuclease’s unintended expression in gene drive applications aimed at suppressing populations of *A. gambiae*, a major malaria vector in Africa. We also explored the feasibility of using these promoters for X-shredding applications, which have thus far relied solely on the *ß2-tub* promoter^13–16,24^.

In summary, we found that both *spo11* and *vasa1* promoters can induce high levels of homing and sex distortion in the progeny of carrier males, a feature that, to our knowledge, has never been reported.

Regarding the *spo11* promoter, our observations indicate that the expression of the Cas9 endonuclease was effectively restricted to the mosquito germline. First, neither maternal nor paternal Cas9 deposition into the embryos was detected (Supplementary Figs. 8 and 9). Furthermore, the absence of sexual mosaicism in the *spo11*/gRNA^*dsx*^ trans-heterozygous females, along with the lack of fertility cost in the *spo11*/gRNA^X^ trans-heterozygous individuals, supports strong evidence that the *spo11* promoter does not exhibit detectable somatic leakiness. This finding identifies an additional promoter for expressing endonucleases, which could prove helpful in genetic strategies to overcome efficiency limitations caused by their misexpression^37^.

Interestingly, we found that the *vasa1* promoter is not male-specific, unlike a previous study in which a fluorescent reporter under the same regulatory sequences was detected exclusively in males^35^. We hypothesise that the discrepancy between the two studies may be due to differences in the transgenes used; while the earlier study employed GFP^35^, we used the Cas9 endonuclease, which may exhibit distinct expression levels or detection sensitivities. The absence of additional studies investigating these regulatory elements currently limits broader cross-experimental comparisons.

The *vasa1* promoter exhibited some somatic leakiness, resulting in sexual mosaicism and sterility in trans-heterozygous *vasa1*:Cas9/gRNA^*dsx*^ female individuals (Fig. 2h–I and Supplementary Fig. 3). However, this was less pronounced than in female mosquitoes where a *vasa2* promoter was employed to express a Cas9 targeting the same locus^42^. Moreover, when using the *vasa1* promoter, no Cas9 deposition was detected, contrary to what has been widely observed for nucleases driven by the *vasa2* promoter^9,37,42^ (Supplementary Figs. 8 and 10). This is also evidenced by the different phenotypes observed when *vasa1* or *vasa2* promoters were tested with the gRNA^X^, which targets the X-linked 28S rDNA locus. While trans-heterozygous adult individuals were found at expected rates using the *vasa1* promoter, we could not recover any when the *vasa2* promoter was employed. This is likely due to Cas9 deposition and somatic expression driven by the *vasa2* promoter, which may have resulted in X chromosome shredding in somatic cells, leading to embryonic or larval lethality (Supplementary Fig. 11).

Interestingly, unlike the *ß2-tub* promoter, which is exclusively expressed in males, *vasa1* and *spo11* are expressed in both sexes, allowing the investigation of inducing X-shredding during female gametogenesis.

Significant cellular damage was induced in these individuals when the *vasa1* promoter was employed, ultimately leading to ovarian underdevelopment and sterility (Fig. 3e, f). This phenotype is similar to the one observed using the *zpg* promoter, expressed early during gametogenesis (Supplementary Fig. 1). Conversely, when the *spo11* promoter was used, females exhibited full fertility (Fig. 3g, h). Sequencing of the rDNA target site revealed little or no signs of mutagenesis, suggesting that either (i) the X chromosomes are not accessible within the timeframe during which *spo11* mediates expression of the Cas9 endonuclease, (ii) the X chromosomes are cleaved and repaired through HDR, possibly facilitated by the presence of the homologous X chromosome and/or internal recombination utilising rDNA repeats as homologous sequences, or (iii) the levels of Cas9 are insufficient to shred both X chromosomes while being adequate to induce X-shredding in males, which have only one X chromosome. Although the underlying mechanisms remain unclear, these findings highlight the complex interplay between X chromosome accessibility and DNA repair during female gametogenesis, which will require further investigation.

Leveraging the unprecedented ability of *vasa1* and *spo11* promoters to efficiently bias both Mendelian inheritance and sex ratio, and inspired by the previously developed SDGD, we developed an SDMD employing a single promoter, *vasa1*, and a single nuclease, Cas9, to efficiently combine homing and sex distortion. By featuring a simplified design containing minimal components, the SDMD system may offer improved molecular and evolutionary stability.

For instance, in a scenario where the sex distorter component is lost, the sterility of SDMD female escapees would prevent the positive selection of such a mutated genetic construct^24^. While the current SDMD design did not allow for testing the effect of the gRNA^*dsx*^ alone on female sterility, the strong fitness costs observed in the trans-heterozygous *vasa1*:Cas9_b/gRNA^*dsx*^ females suggest that Cas9 activity at the *dsx* locus severely compromises their fertility.

To further ensure the sterility of such female escapees, independently of the sex distorter component, an SDMD system could be engineered into loci known to confer dominant female sterility. For instance, a recently characterised 11 bp deletion at the gRNA^*dsx*^ target site functions as a dominant negative allele, causing heterozygous females to develop as sterile intersex individuals^20^. Such alleles could be strategically leveraged to induce sterility, irrespective of somatic leakiness or X-shredding.

Importantly, SDMD systems are predicted to exhibit greater resilience to the emergence and spread of target site resistance compared to SDGD designs, due to the sterility imposed on female carriers (Figs. 1 and 6). As functional resistant alleles are positively selected in females, but not in males, ensuring the sterility of the female escapees significantly reduces the effectiveness of selection for resistance, leading to a much prolonged period of time over which the population is suppressed.

No functional resistance has been detected in any previous cage trials of *dsx*-targeting gene drives, whether or not a sex-distorter element was included, across both small- and large-scale experiments^9,11,12,24,43^. These results suggest that further cage trials at this locus are unlikely to yield additional insights into resistance evolution under laboratory conditions, where population sizes are inherently constrained. Nonetheless, the absence of detectable resistance in laboratory settings does not preclude its emergence following field release, highlighting the importance of the improvements achieved with the SDMD design. To more accurately anticipate the dynamics of resistance evolution, approaches extending beyond conventional cage experiments will be required, with mathematical modelling playing a central role in this effort. In addition, such modelling could incorporate local, ecological and epidemiological factors^44^ to evaluate the precise impact of quantitative reductions in the number of biting females on the burden of malaria.

By employing a CRISPR-Cas9-based X-shredding approach instead of the *I-PpoI* endonuclease, our design encompasses greater versatility than the previously developed SDGD. This allows for the potential to combine homing and X-shredding in a wider range of insect species^15,16^ and to target polymorphisms unique to genetically distinct populations^45^.

The SDMD system presented here serves as a proof-of-principle for combining homing and X-shredding via a single promoter and nuclease, simplifying previous SDGD designs and advancing resistance management. However, further optimisation of this construct is needed to enhance its efficacy.

For example, the analysis of the progeny that did not inherit the SDMD construct revealed that about 95% of the alleles were unmutated, suggesting that reduced homing efficiency may result from suboptimal Cas9 activity levels or timing, rather than a preferential use of NHEJ over HDR (Supplementary Figs. 6 and 7). Modifying the regulatory elements of *vasa1* or incorporating transcriptional enhancers^46^ may shed light on whether tuning Cas9 expression can improve homing efficiency without incurring fitness costs in males.

Consistent with our findings and previous studies, homing and sex distortion efficiency are highly locus-dependent^13,37^. Targeting other female fertility genes may optimise both processes, improving the suppression potential of this approach.

In addition, incorporating multiplexed gRNA arrays represents another avenue to reduce the emergence of resistant alleles^9^.

To fully evaluate the efficacy of any improved SDMD systems, additional tests on transgenic males, such as flight ability, could be performed to provide a more comprehensive assessment of their fitness.

In summary, the promoters examined in this study provided valuable insights into mosquito gametogenesis and contributed to the advancement of an SDMD technology. While further optimisation of the current design will be advised to evaluate its potential for population suppression fully, the SDMD approach holds considerable promise for the genetic control of the malaria vector, *A. gambiae*.

## Methods

### Mosquito rearing

All the experiments were conducted on the *A. gambiae* G3 strain (MRA-112), obtained from the Malaria Research and Reference Reagent Resource Centre (MR4). The strain was originally isolated from the Gambia in 1975. All the mosquitoes were maintained in a Containment Level 2 insectary at Imperial College London in a 12 h light/dark cycle at 27 °C ± 1 and 70% ± 5 relative humidity. Aquatic stages were reared in trays filled with 0.5% salt water and fed on fish food (Nishiokoi). Adult stages were fed on 5% glucose solution, and females were fed on cow blood through a membrane feeding system (Hemotek Ltd).

### Plasmids construction

All the primers used for plasmid generation are listed in Supplementary Table 3. PCRs were performed using Phusion High-Fidelity PCR master mix with HF buffer (Thermo Scientific™, UK) and amplicons purified using Wizard® SV gel and PCR clean-up start-up kit (Promega, UK). Constructs cloned using Golden Gate assembly (T4 DNA ligase and buffer New England Biolabs, UK; FastDigest Eco31I Thermo Scientific™, UK) were transformed in Invitrogen™ One Shot™ TOP10 Chemically Competent *E. coli* (Fisher Scientific, UK). NEB 5-alpha Competent *E*. *coli* #C2987 (New England Biolabs, UK) were used to transform plasmid assembled through Gibson assembly (Gibson Assembly Master Mix NEB #M5510, New England Biolabs, UK). To isolate the putative regulatory sequences of *spo11*, 2878 bp upstream of the translation start site and 973 bp downstream of the stop codon were amplified from gDNA extracted from wild type *A. gambiae* G3 strain using *spo11_*P_F/R and *spo11_*T_F/R primers to serve as promoter and terminator, respectively. The isolated regulatory sequences of *spo11* were used to generate the p_*spo11*:Cas9 construct through Gibson assembly with the hCas9-F2A-mCherry cassette, the *3xP3*:GFP cassette and PiggyBac inverted repeats, each amplified from previously developed plasmids using primers designed to include overhangs. To clone the p_*vasa1*:Cas9 construct, the *vasa1* promoter and terminator were isolated from the previously developed Vas1GFP plasmid^35^, and assembled with the hCas9-F2A-mCherry-*3xP3*:GFP-PiggyBac cassette, through Gibson assembly. To generate the p_SDMD construct, the *vasa1* promoter-hCas9-*vasa* terminator cassette was first excised from a previously developed construct using AgeI and SgsI restriction enzymes. This was ligated into the backbone of the p165^10^ plasmid, which was also digested with the AgeI and SgsI restriction enzymes, leading to the switch of the *vasa2*:hCas9 transcription units with the *vasa1*:hCas9 cassette. The p165 plasmid also contains a U6:gRNA cassette designed for inserting the spacer of the gRNA of interest via Bsa-I-mediated Golden Gate, through which the gRNA^X^ spacer (rDNA 5’- GGGTACAAGCTTGCGTACGT-3’)^14^ was inserted. The resulting plasmid was linearised through restriction digestion using the SfaI enzyme, followed by dephosphorylation through Thermosensitive Alkaline Phosphatase (FastAP^TM^, Thermo Scientific, UK). Such a linearised vector was used as a template for Gibson assembly with a second U6:gRNA cassette targeting *dsx* (5′-GTTTAACACAGGTCAAGCGG-3’), amplified from the p16510 plasmid^11^ with primers containing overhangs for cloning.

### Generation and characterisation of transgenic strains

All the transgenic strains were generated following a previously established protocol for microinjection of *A. gambiae* embryos^47^. To generate the *spo11*:Cas9 and *vasa1*:Cas9 strains, freshly laid *A. gambiae* G3 embryos were injected with 200 ng/μl of a *vasa2*-transposase helper plasmid and 100 ng/μl of p_*spo11*:Cas9 and p_*vasa1*:Cas9 plasmids, respectively. To generate the SDMD strain, 200 ng/μl of a *vasa2*-φC31 integrase helper plasmid was co-injected with 250 ng/μl of the p_SDMD plasmid into embryos from the Ag(KFS)2 docking strain, which harbours a *3xP3*:GFP cassette into the female-specific intron 4—exon 5 boundary of *dsx*, flanked by two AttP sites for integrase-mediated recombination. All G_0_ survivors were separated according to the presence or absence of transient expression of the fluorescent marker in the terminal part at the L1 larval stage and reared to the adult stage. Three days post-eclosion, transient and non-transient G_0_ individuals were crossed to wild types, and blood feeding was provided after 5 days of mating. The G_1_ progenies of such crosses were screened for expression of the fluorescent marker in the nervous tissue, specifically the optic lobe and dorsal ganglia, where *3xP3* promotes expression of either GFP (p_*spo11*:Cas9 and p_*vasa1*:Cas9) or RFP (p_SDMD). Recordings of embryo microinjections for each construct are presented in Supplementary Table 4. Transgenic G_1_ male and female pupae were separated and crossed to wild types, followed by single depositions and screening of G_2_ to select single insertion events according to the inheritance of the fluorescent marker. To characterise the genomic location of the transgene in the *spo11*:Cas9 and *vasa1*:Cas9 strains, inverse PCR was performed using iPCR primers shown in Supplementary Table 3 and the genomic location of the transgene for each strain is shown in Supplementary Table 1. To confirm the insertion locus of the transgene in the SDMD strain, PCR were performed with primers *dsx*_e4_F2/gRNA^X^_R1 (5’ flanking-transgene) and RFP_F2/*dsx*_e5_R2 (transgene-3’ flanking) (Supplementary Table 3), followed by gel extraction of the amplicons and Sanger sequencing.

### Phenotypic assays

Fertility assessment of *spo11*:Cas9 and *vasa1*:Cas9 strains was performed by crossing 50 heterozygous males and females to 50 wild types. Control crosses were set up using the same number of male and female wild types, following the same set-up of the experimental groups. The fertility assessment of the SDMD strain was performed only on heterozygote individuals inheriting the construct paternally since the heterozygous females were sterile. For each assay, mosquitoes were crossed for 5 days and blood-fed by Hemotek for 30 min. After 3 days, females were put in cups for egg laying, and eggs and larvae from each female were counted individually to measure the fecundity. Statistical analysis of eggs and larval output was performed using Kruskal–Wallis and Mann–Whitney tests, while Fisher’s exact test was used to determine significant differences in the hatching rate in comparison to wild-type controls. All the statistical tests were performed using GraphPad Prism 10.6.0 (796) Macintosh Version by Software MacKiev © 1994–2025 GraphPad Software, LLC.

### Assessment of homing

Genetic crosses and single depositions were set up as described above using 50 male and female individuals. Homing levels were assessed by scoring the RFP inheritance in the progeny of *spo11*:Cas9/gRNA^*dsx*^ and *vasa1*:Cas9/gRNA^*dsx*^ trans-heterozygous or SDMD heterozygous males and females crossed to wild types. Larvae were screened within 24 h of hatching, at the L1 stage. For each cross, fertility was assessed by counting eggs, larvae and calculating the hatching rate in comparison to the control. Statistical analysis of eggs and larval output was performed using Kruskal–Wallis and Mann–Whitney tests. Statistical analysis of homing rate in *spo11*:Cas9/gRNA^*dsx*^ and *vasa1*:Cas9/gRNA^*dsx*^ was conducted using Fisher’s exact test on the total fraction of progeny inheriting RFP marker in comparison to gRNA^*dsx*^ controls. All the statistical tests were performed using GraphPad Prism 10.6.0 (796) Macintosh Version by Software MacKiev © 1994-2025 GraphPad Software, LLC.

### Assessment of sex ratio distortion

To assess the ability of *spo11*:Cas9, *vasa1*:Cas9 and SDMD to skew the sex ratio of the progeny toward males, genetic crosses and single depositions were performed as described above, followed by rearing of the progeny until the adult stage, when males and females were counted. Statistical analysis of eggs and larval output was performed using Kruskal–Wallis and Mann–Whitney tests. Statistical analysis of sex ratio distortion was conducted using Fisher’s exact test on the total fraction of male progeny in comparison to wild-type controls. All the statistical tests were performed using GraphPad Prism 10.6.0 (796) Macintosh Version by Software MacKiev © 1994–2025 GraphPad Software, LLC.

### Cas9 deposition testing

To test whether Cas9 endonuclease is deposited in the embryo when transcribed under the regulatory sequences of *spo11* and *vasa1*, we crossed *spo11*:Cas9_a and *vasa1*:Cas9_b with the gRNA^*dsx*^ strain and selected the gRNA^*dsx*^-only progeny for further analysis, including fertility assessment, shadow homing analysis and pooled amplicon sequencing. Since they do not genetically encode for Cas9, the only source of mutations at the wild type allele at the *dsx* target site would be parental deposition of the nuclease, which would form an active CRISPR-Cas9 complex with the gRNA^*dsx*^ and create double strand breaks at the target site. If repaired through NHEJ, these mutations could create mosaicism at the intron 4–exon 5 boundary of *dsx*, possibly impacting the fertility of gRNA^*dsx*^-only females. Fertility assessment was performed by crossing 30 gRNA^*dsx*^-only females to wild-type males and counting of eggs and larval progeny from single depositions. gRNA^*dsx*^-only male siblings from the same crosses were also tested as a control, since their fertility is not expected to be impacted by mutagenesis at a female-specific locus, as well as gRNA^*dsx*^ strain, which was never exposed to any source of Cas9. Progeny was also screened for *3xP3*:RFP, the fluorescent marker included in the transgene of the gRNA^*dsx*^ strain, to investigate HDR of the cuts induced by the deposited Cas9, in a process defined as ‘shadow homing’.

### Amplicon sequencing

Genomic DNA extraction was performed on either single or pooled samples of mosquitoes (Wizard Genomic DNA purification kit, Promega, UK). Amplicon sequencing on single individuals was performed on three trans-heterozygous *vasa1*:Cas9/gRNA^*dsx*^ females exhibiting sexual mosaicism to assess the level and the nature of mutagenesis occurring at the *dsx* target site, responsible for the mosaic phenotype. Pooled amplicon sequencing was employed to study the mutagenesis across a larger number of samples to assess the proportion and the nature of the EJ events following Cas9 activity, for instance, due to EJ during male gametogenesis. The number of individuals pooled for each experiment is indicated in the corresponding figure legend or text. Two replicates were set up for PCR reactions using 100 ng of gDNA using KAPA HiFi HotStart ReadyMix (Roche) in 50 ml reactions. One replicate was run for x40 amplification cycles and loaded on a 1× agarose gel to verify the presence of the amplicon of the expected size. The other replicate was run under non-saturating conditions (×20 cycles), and amplicons were purified using Wizard® SVGel and PCR Clean-Up System (Promega). A 356 bp and 333 bp loci spanning the *dsx*F and rDNA target sites, respectively, were amplified using Illumina-AmpEZ-4050-F/R and Illumina-AmpEZ-rDNA-F/R primers. Amplicon sequencing was performed through next-generation sequencing Amplicon-EZ (Genewiz). Data were analysed using CRISPResso2 software v2.0.29.

### DAPI staining of reproductive organs and microscopy

To assess the phenotype of the gonads of *vasa1*:Cas9_b/gRNA^X^ and *zpg*:Cas9/gRNA^X^ transheterozygous (Fig. 3f and Supplementary Fig. 11), as well as SDMD female escapees (Fig. 4d), the ovaries of 2-day-old unfed mosquitoes were dissected in 1× PBS and transferred to an embryo dish containing a fixative solution (4% formaldehyde in 1× PBS for testes or 4% formaldehyde in 0.1% Tween 1× PBS for ovaries). Following a 30 min incubation at room temperature, dissected gonads were washed 3 times with a 0.1% Tween 1× PBS solution for 15 min each. Fifteen microliters of Prolong Antifade gold + DAPI were used to mount testes and ovaries in a microscope slide, covered with a coverslip and sealed with Cytobond rubber cement. Slides were incubated at 4 °C for at least 24 h before analysing them at a confocal microscope using either Leica SP8 inverted or Leica SP8-STELLARIS 5 Inverted confocal microscopes (Imperial College London FILM facility). A 63x oil immersion objective was used for image acquisition using tile scanning for ovaries and Z-stack for testes. Images were processed using Fiji software^48^.

### Mathematical modelling

We use a stochastic density-dependent model with discrete time and non-overlapping generations. We consider 4 alleles: wild type (W), drive (D), non-functional resistant (R2) and functional resistant (R1). The model tracks the 10 possible genotypes for both males and females {WW, WD, WR2, WR1, DD, DR2, DR1, R2R2, R2R1, R1R1}. Each female mates with a male whose genotype is sampled using a multinomial distribution with probabilities equal to the males’ genotype frequencies, i.e. males are not limited in their mating, while females mate only once. Males and females form gametes with probabilities given in Supplementary Fig. 13 and Supplementary Table 5 following the multinomial distribution, and the genotypes of the offspring are assigned accordingly. Parameter values for the generalised modelling are given in Supplementary Table 6, and those for the construct-specific modelling in Supplementary Table 7. The sex of the offspring is assigned independently using the Binomial distribution. It is assumed that only males carrying the D allele have progeny with $m:(1-m)$ ratio of males to females; in case only females or neither male nor female carry gene drive, the ratio is $0.5\,:0.5$. Each female produces $f$ eggs on average, sampled from a Poisson distribution. The intrinsic rate of increase of the population ${R}_{m}=\frac{f}{2}$, and we use a typical value of ${R}_{m}=6$ in the simulations. We model juvenile density-dependent mortality based on the Beverton–Holt model^49^, with the probability of surviving to adulthood of the hatchlings equal to $\frac{\alpha }{\left(\alpha+{N}_{h}\right)}$, where ${N}_{h}$ is the total number of hatchlings in the generation and $\alpha$ is chosen to give the desired initial pre-release population size (*N* = 10^15^).

Fitness costs are modelled as a decrease in fecundity relative to the wild type. There are assumed to be no fitness costs for carrying a non-functional resistant allele for any construct. All males are assumed to have fitness 1. In all constructs, functional resistance has no fitness costs^9^, while non-functional resistant alleles block the drive but impose significant fitness costs. The outputs were averaged over 100 simulations.

### Statistics and reproducibility

The statistical tests and sample sizes used in each experiment are described in the corresponding figures, figure legends and methods. Sample size was chosen consistent with the previous literature reporting similar assays and maximised within the feasibility of performing biological assays with live insects. No data were excluded from the analyses.

### Reporting summary

Further information on research design is available in the Nature Portfolio Reporting Summary linked to this article.

## Supplementary information

Supplementary Information

Reporting Summary

Transparent Peer Review file

## Source data

Source Data

## Data Availability

Amplicon sequencing data generated and analysed in this study are available in the Sequence Read Archive (SRA) with the accession code PRJNA1285401 [https://www.ncbi.nlm.nih.gov/bioproject/PRJNA1285401]. Source data are provided with this paper.

## Code availability

Code used for the simulations performed in this study is fully available on GitHub: [https://github.com/OksanaVertsimakha/SDMD], under a GNU General Public Licence 3.0 (GPL-3.0), 10.5281/zenodo.18548498.
