# Supplementary material for: Sex distorter male drive for resistance-resilient population control of the human malaria vector Anopheles gambiae
Source: Nat Commun. 2026 Apr 11;17:5109. doi: 10.1038/s41467-026-71627-1 (PMC13246785; doi:10.1038/s41467-026-71627-1)
Supplement: Supplementary file 1 — Supplementary Information [file 41467_2026_71627_MOESM1_ESM.pdf]

Supplementary Figures

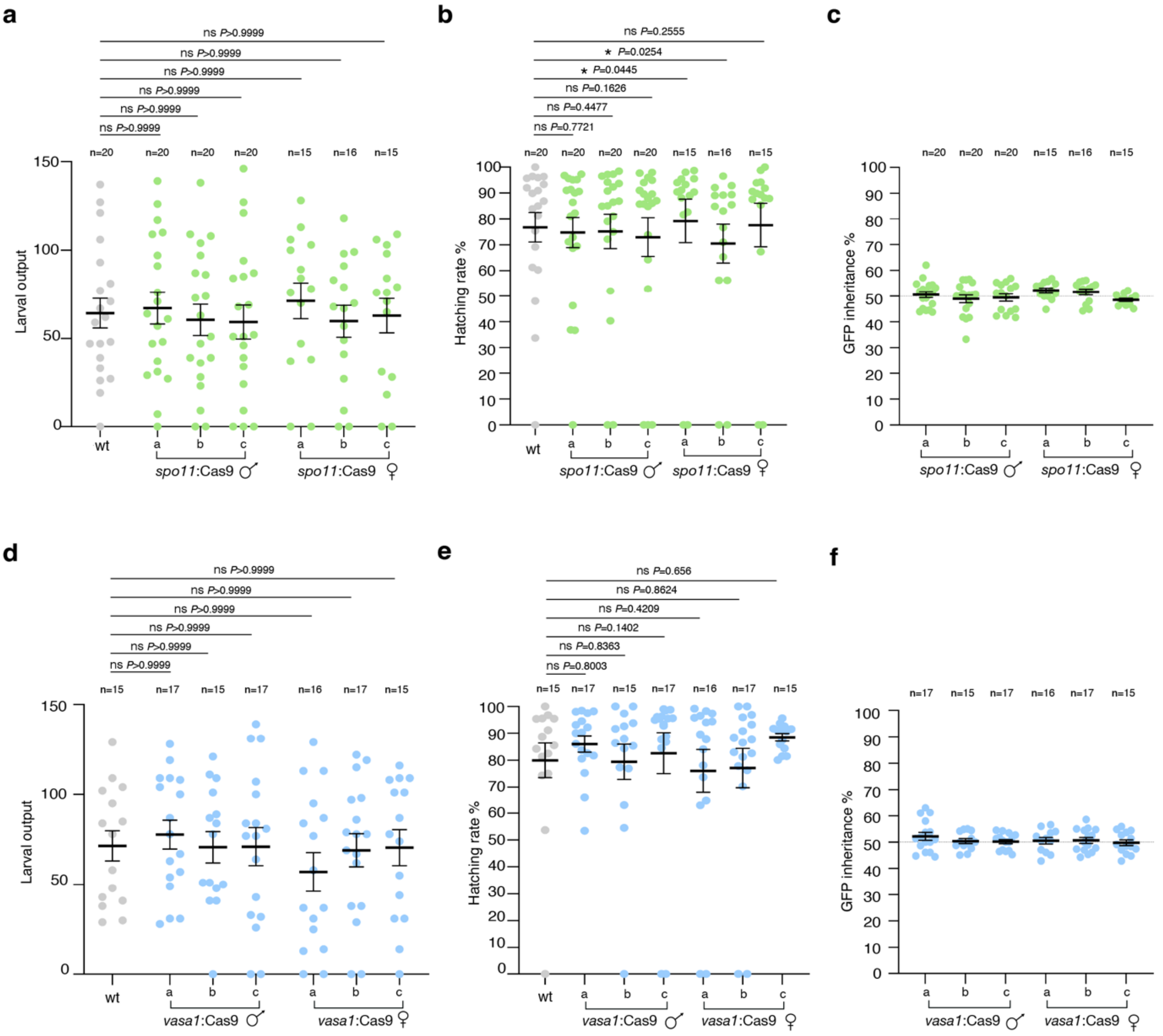

Supplementary Fig. 1 Fertility assessment of *spo11*:Cas9 and *vasa1*:Cas9 strains. a,d Larval output and **b,e** hatching rate were measured to assess the fertility of the *spo11*:Cas9 (blue) and *vasa1*:Cas9 (green) strains in comparison to wild types (grey). Heterozygous males or females were crossed *en masse* with their wild type counterparts and females individually separated for eggs laying. Progenies from each group were individually counted at egg and larval stages to calculate the hatching rate. **c,f** *3xP3*:GFP inheritance in each progeny was scored to assess the number of integration events in the genome, where 50% of inheritance represents the insertion

of the transgene in one single locus. Dots represent the progeny of a single female mosquito. Thick horizontal lines with error bars indicates the arithmetic mean of larval output and homing rates and s.e.m., respectively. Statistical analysis of larval output was performed using Kruskal-Wallis test followed by two-sided Dunn's multiple comparison test with the wild type control. Statistical analysis of hatching rate was performed by comparing the total number of eggs and larvae in the experimental group versus their respective wild type controls (Fisher's exact test, two-sided). P values and sample size (n) are indicated in the figure on top of each data group. Source data are provided as a Source Data file. Graphs and statistical analysis were generated using GraphPad Prism (v10.6.0).

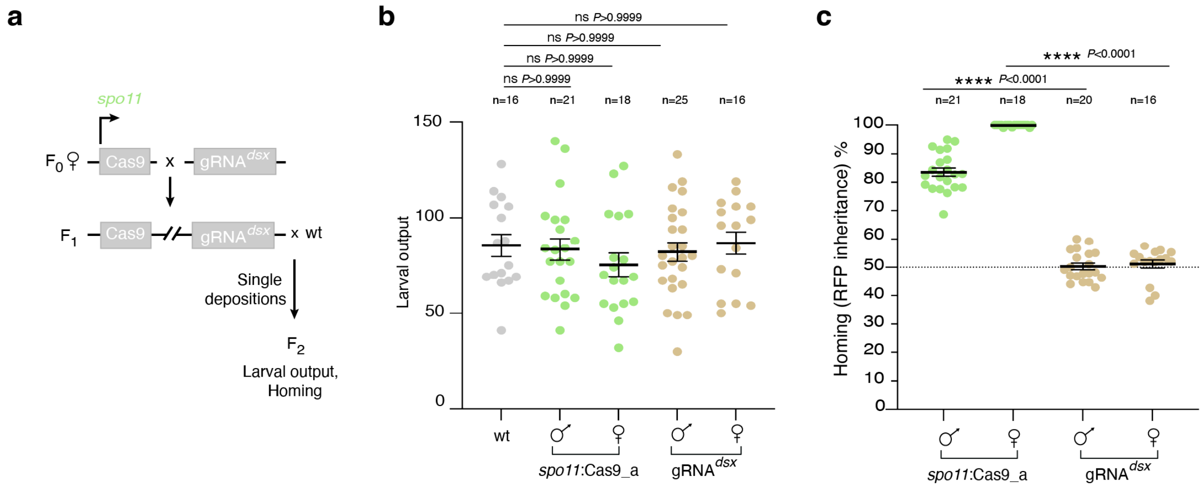

Supplementary Fig. 2 Homing assessment in the *spo11*:Cas9_a/gRNA^{dsx} trans-heterozygotes when Cas9 is inherited maternally. a Schematic of the genetic crosses to assess the fertility (**b**) and homing rates (**c**) of *spo11*:Cas9/gRNA^{dsx} trans-heterozygous males and females (green) inheriting the Cas9 maternally. Control groups include wild type (grey) and the gRNA^{dsx} strain (beige). Dots represent the progeny of a single female mosquito. Thick horizontal lines with error bars indicates the arithmetic mean of larval output and homing rates and s.e.m., respectively. Statistical analysis of larval output was performed through Kruskal-Wallis test followed by two-sided Dunn's multiple comparison test with the wild type control. Statistical analysis of homing rate was performed by comparing the total number of RFP+ and RFP- individuals in the experimental group versus the wild type controls (Fisher's exact test, two-sided). P values and sample size (n) are indicated in the figure on top of each data group. Source data are provided as a Source Data file. Graphs and statistical analysis were generated using GraphPad Prism (v10.6.0).

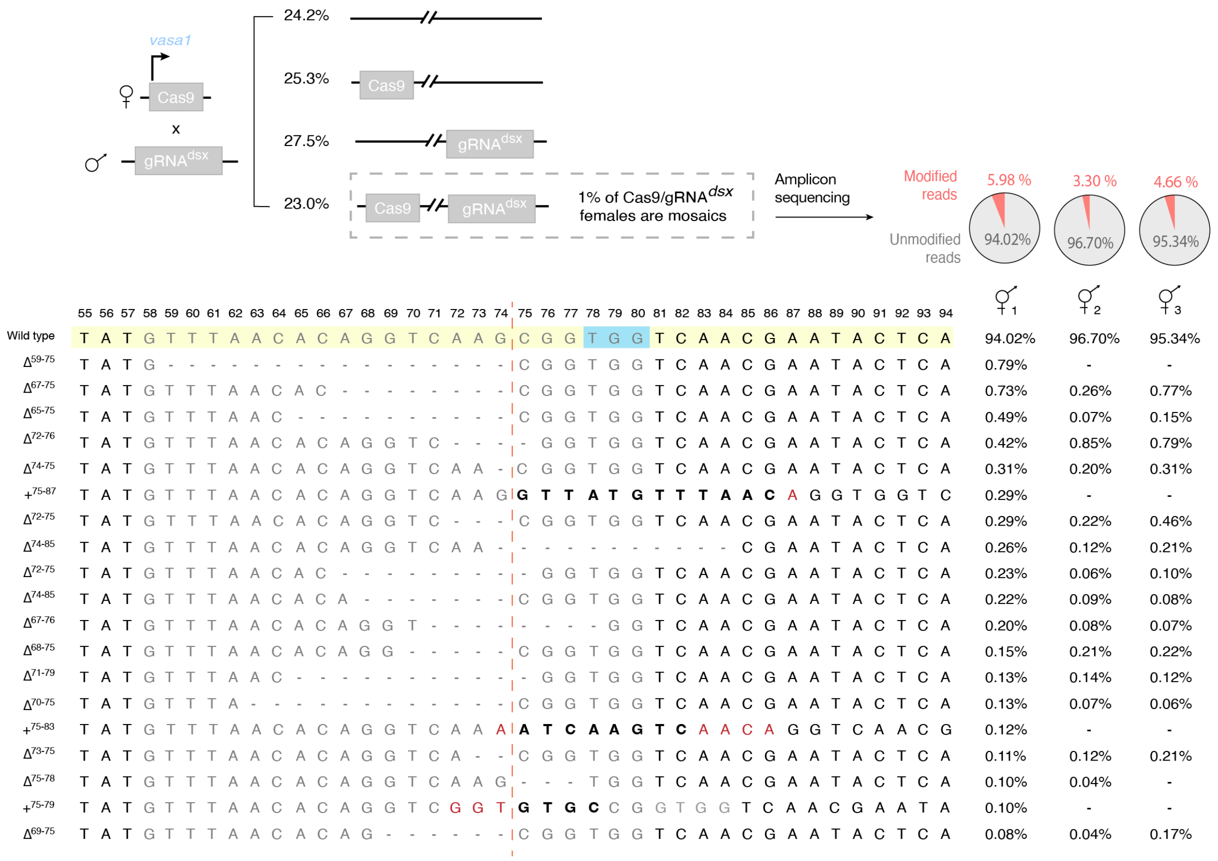

Supplementary Fig. 3 Mutagenesis at the *dsx* target site in the trans-heterozygous *vasa1*:Cas9/gRNA^{dsx} female individuals. Schematic representation and proportion of the different genotypes in the progeny of the genetic cross between *vasa1*:Cas9_b females and gRNA^{dsx} males (n=2600). Approximately 1% (n=3) of the *vasa1*:Cas9/gRNA^{dsx} females showed sexual mosaicism, exhibiting intermediate males and female features at the external pupae genitalia, indicated in the figure with the ⚥ symbol. Amplicon sequencing was performed on these three adult individuals displaying sexual mosaicism for which the proportion of the mutated reads is illustrated in their respective pie charts. The sequences of the indels or substitutions observed with a frequency above 0.1% are shown. The wild type sequence is highlighted in yellow. The 20 nt comprising the gRNA spacer are depicted in grey, and the PAM is

highlighted in blue. The red dashed line represents the cut site. Deletions are indicated with dashes at the corresponding nucleotide and insertions in bold.

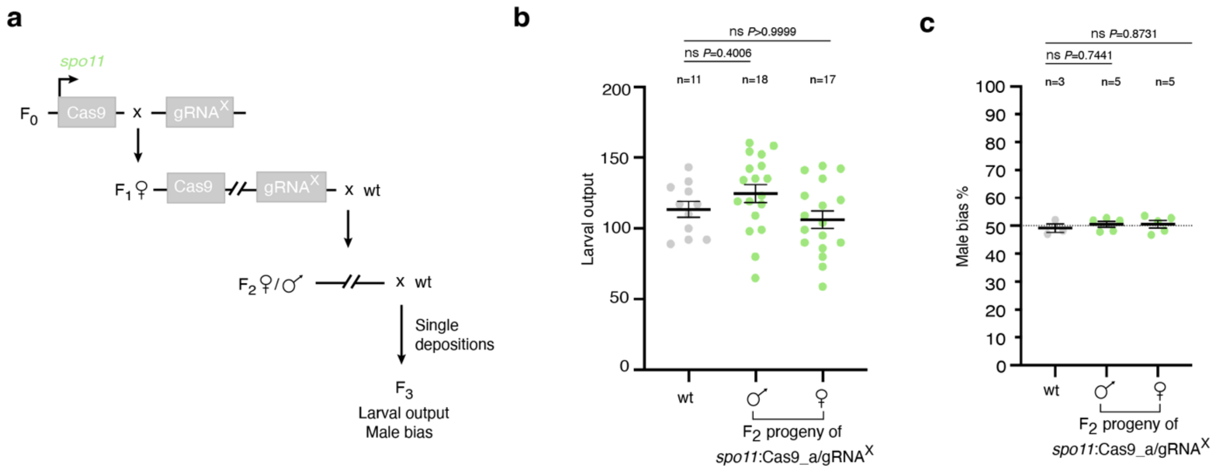

Supplementary Fig. 4. Fertility assessment of the non-transgenic male and female progeny (F_2) of *spo11*:Cas9/gRNA^X trans-heterozygous females. a Schematics of the genetic crosses performed to investigate the fertility of the progeny of *spo11*:Cas9/gRNA^X trans-heterozygous females (green), in which the X chromosomes are exposed to the activity of the Cas9 endonuclease directed by the gRNA^X to the X-linked 28S rDNA repeats. F_2 progeny inheriting neither the Cas9 nor the gRNA^X was outcrossed to wild types. Females were provided with a blood meal and separated for egg laying. A wild type group (grey) was included as control. **b** Larval output was assessed to measure fertility. Statistical analysis of larval output was performed using Kruskal-Wallis test followed by two-sided Dunn's multiple comparison test with the wild type control. **c** Some progenies were reared to the adult stage to assess the sex ratio. Statistical analysis of male bias was performed by comparing the total number of males and females in the experimental group versus their respective

wild type controls (Fisher's exact test, two-sided). P values and sample size (n) are indicated in the figure on top of each data group. Source data are provided as a Source Data file. Graphs and statistical analysis were generated using GraphPad Prism (v10.6.0).

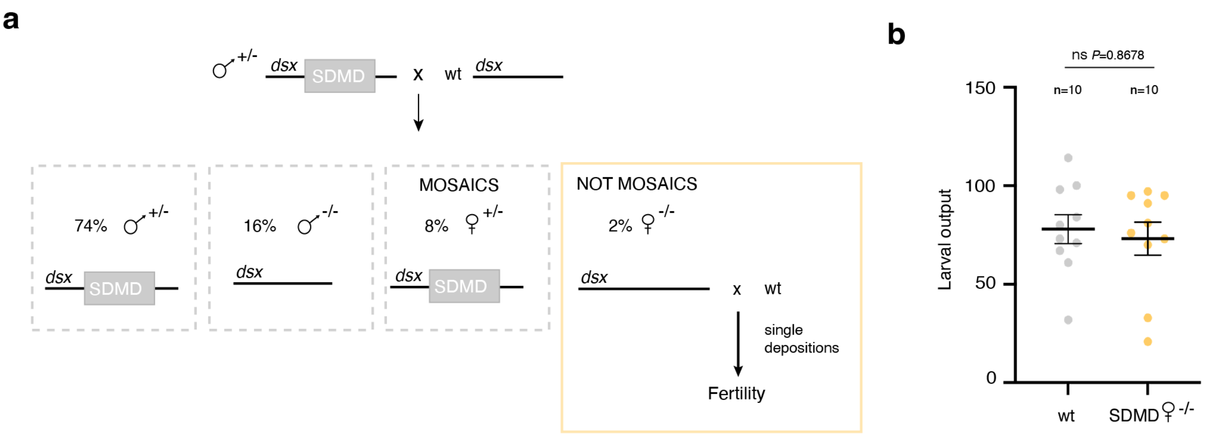

Supplementary Fig. 5. SDMD escapee females not harbouring the transgene do not exhibit sexual mosaicism and are fertile. a Schematic of the genotypes generated by SDMD heterozygote males crossed to wild type females, showing their relative frequency (n=500). The SDMD escapee females not inheriting the transgene (n=10) were outcrossed to wild type males and (**b**) their fertility assessed by larval counting (yellow) in comparison to wild type (grey). Statistical analysis of larval output was performed using two-sided Mann Whitney test. P values and sample size (n) are indicated in the figure above each data group. Source data are provided as a Source Data file. Graphs and statistical analysis were generated using GraphPad Prism (v10.6.0).

Investigating the origin of end-joining mutations in the negative progeny of SDMD heterozygous males

The negative progeny of heterozygous SDMD males was analysed for mutations at the *dsx* target sites through pooled amplicon sequencing (n=100), revealing that a small proportion of reads were mutagenized (Supplementary Fig. 6). Such mutations could have originated either in the parental germline following Cas9 activity or in the embryo due to endonuclease deposition. To discriminate between these two events, we leveraged the availability of the Ag(KFS)2 docking strain, which harbours a *3xP3*:GFP cassette at the gRNA^{dsx} target site. A first genetic cross was performed between heterozygous SDMD males and Ag(KFS)2 females, and the progeny inheriting either only the Ag(Dsx) allele or both the SDMD and the Ag(KFS)2 alleles were separated for further analysis. At first, pooled amplicon sequencing (n=50) performed at the gRNA^{dsx} target site on the cohort of individuals inheriting only the Ag(KFS)2 construct revealed that about 14% of the total reads were mutated (Supplementary Fig. 7a). To discriminate between NHEJ events occurring in the parental germline and those in the embryo due to Cas9 deposition, a second genetic cross was performed on the male individuals inheriting both the SDMD and the Ag(KFS)2 alleles to wild type females. Their GFP-only, i.e. those inheriting only the Ag(KFS)2 construct, was assessed for mutagenesis at the gRNA^{dsx} target site through pooled amplicon sequencing (Supplementary Fig. 7b). As these individuals did not genetically encode for the Cas9 endonuclease, and the gRNA^{dsx} target site in the germline of the SDMD/Ag(KFS)2 parents was not present due to disruption by the SDMD and *3xP3*:GFP cassettes, mutagenesis at the *dsx* target site would reflect paternal deposition of the Cas9. In these individuals, we observed neither sexual

mosaicism nor mutagenesis, suggesting that Cas9 is not paternally deposited in the SDMD strain (Supplementary Fig. 7b).

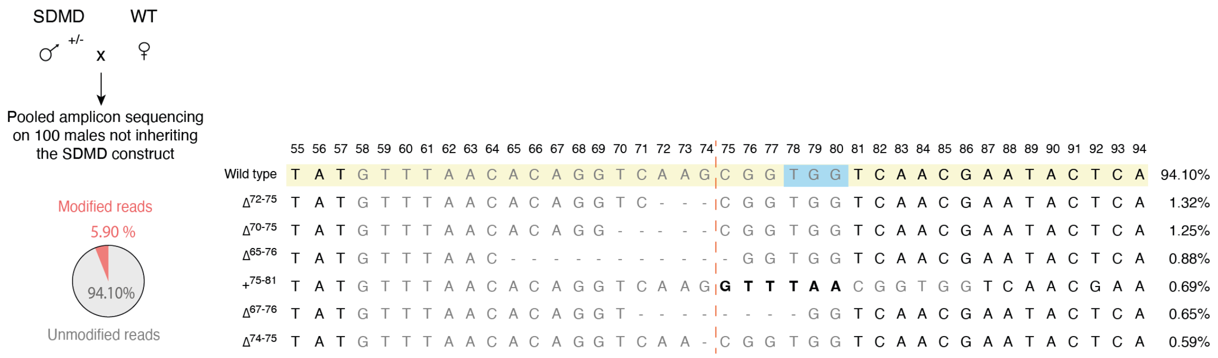

Supplementary Fig. 6. Mutagenesis at the gRNA^{dsx} target site in the SDMD negative male progeny. Schematic illustration of the mutations observed in amplicon sequencing of pooled RFP- progeny (n=100) of SDMD males. Mutations with a frequency above 0.5% are shown which, in the pool of the 100 individuals sequenced, correspond to one mosquito heterozygote for such mutation. The wild type sequence is highlighted in yellow. The 20 nt comprising the gRNA spacer are depicted in grey, and PAM highlighted in blue. The red dashed line represents the cut site. Deletions are indicated with dashes at the corresponding nucleotide and insertions in bold.

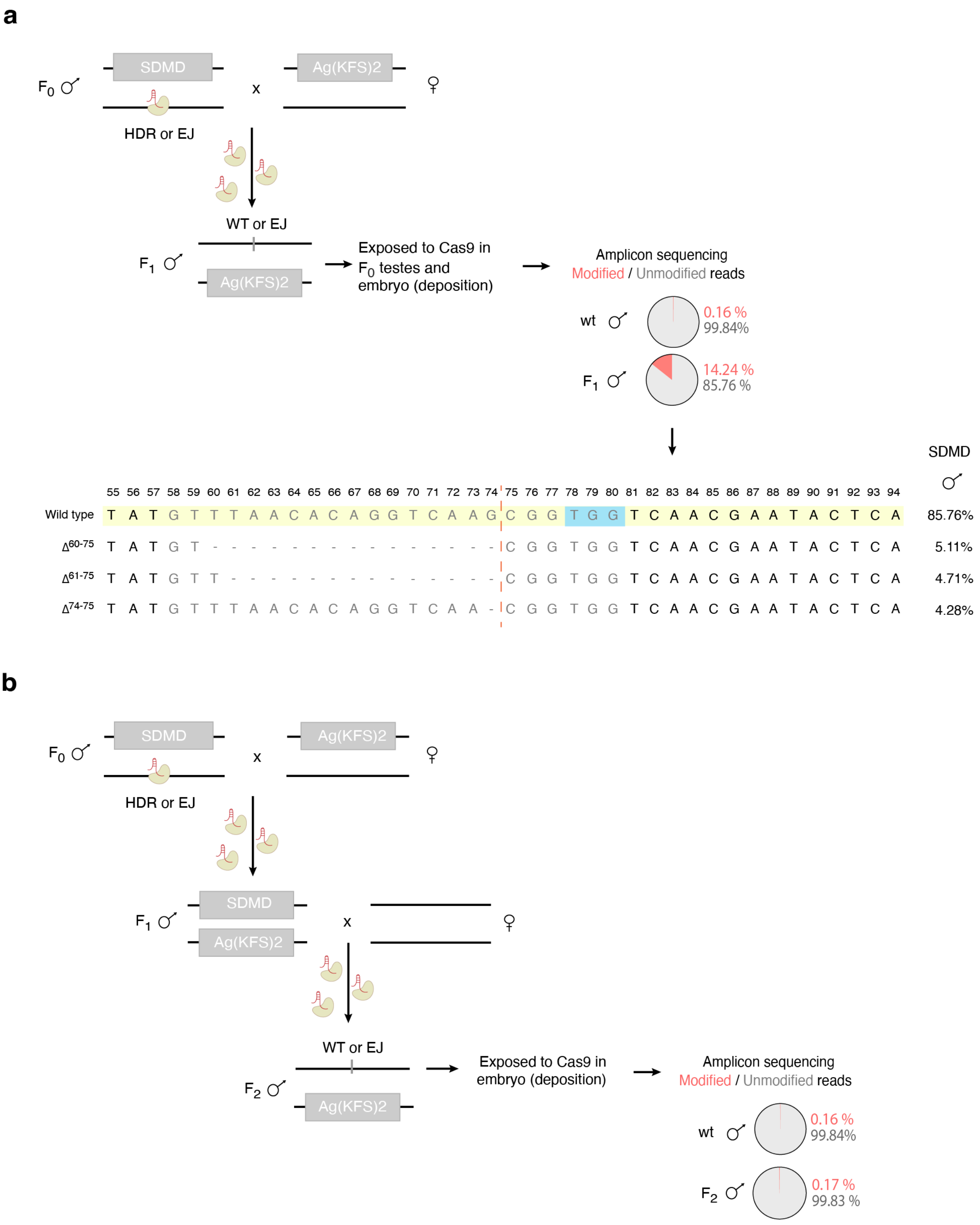

Supplementary Fig. 7 Assessment of the origin of the end-joining mutations in the SDMD strain. **a** Schematic representation of the genetic cross between the SDMD and the Ag(KFS)2 docking strains to assess the rate of end-joining mutations

which could have originated following Cas9 activity either in the parental germline or in the embryo due to endonuclease deposition. F_1 individuals inheriting only the Ag(KFS)2 allele (n=50) were selected for pooled gDNA extraction and amplicon sequencing was performed at the *dsx* target site. The pie charts illustrate the proportion of mutated reads in these individuals (14.24%). The most frequent mutations ($\Delta^{60\text{-}75}$, $\Delta^{61\text{-}75}$, $\Delta^{74\text{-}75}$) corresponding to at least one heterozygote individual for that mutation are shown. The wild type sequence is highlighted in yellow. The 20 nt comprising the gRNA spacer are depicted in grey, and PAM highlighted in blue. The red dashed line represents the cut site. Deletions are indicated with dashes at the corresponding nucleotide. **b** Schematic representation of the genetic cross between the SDMD and the Ag(KFS)2 docking strains to assess the rate of end-joining mutations which could have originated following Cas9 activity in the embryo due to endonuclease deposition. The SDMD/Ag(KFS)2 trans-heterozygous F_1 male progeny was outcrossed to wild type females and F_2 individuals inheriting only the Ag(KFS)2 allele (n=150) were selected for pooled gDNA extraction and amplicon sequencing was performed at the *dsx* target site. The frequency of mutations in each analysed pooled sample is less than the expected frequency of one heterozygote individual, and comparable to the wild type control, suggesting absence of Cas9 deposition in the SDMD strain.

Assessing the propensity of *spo11* and *vasa1* promoters to induce Cas9 deposition

To assess Cas9 deposition, we set up genetic crosses between *spo11:Cas9* or *vasa1:*Cas9 and the gRNA^{dsx} strains to isolate progeny that inherited only the gRNA^{dsx}. In these individuals, deposition of the endonuclease would result in the formation of an active CRISPR-Cas9 complex with the genetically encoded gRNA^{dsx}, enabling cleavage at the *dsx* locus in the embryos.

Such cleavages could occur in both the progenitor cells of somatic tissue, leading to mutations, sexual mosaicism and fitness costs, and in the progenitor cells of the germline. In this case, if the DNA breaks are repaired through HDR, this could result in a biased inheritance of the transgene even in the absence of an encoded nuclease, referred to as a shadow drive[1].

The gRNA^{dsx}-only progeny was analysed for mutagenesis through pooled amplicon sequencing. Additionally, fertility and gRNA inheritance rates were assessed. As a positive control, we used a previously generated *vasa2*:Cas9 strain, as such a promoter has been widely characterised and shown to induce high levels of maternal deposition[2]. In line with this, pooled amplicon sequencing at the *dsx* target site in the gRNA^{dsx}-only progeny of the cross between *vasa2*:Cas9 females and gRNA^{dsx} males, revealed that >99% of the reads were mutated (Supplementary Fig. 8).

Conversely, for both *spo11* and *vasa1* promoters, we observed no significant mutagenesis at the *dsx* locus (<0.3%) of their gRNA^{dsx} -only progeny (Supplementary Fig. 9a-b, 10a-b). Furthermore, no fertility costs were observed in these individuals (Supplementary Fig. 9c-d, 10c-d). Additionally, the absence of shadow homing in these individuals (Supplementary Fig. 9e-f, 10e-f) further suggests that neither *spo11* nor *vasa1* promoters induce Cas9 deposition in the analysed strains.

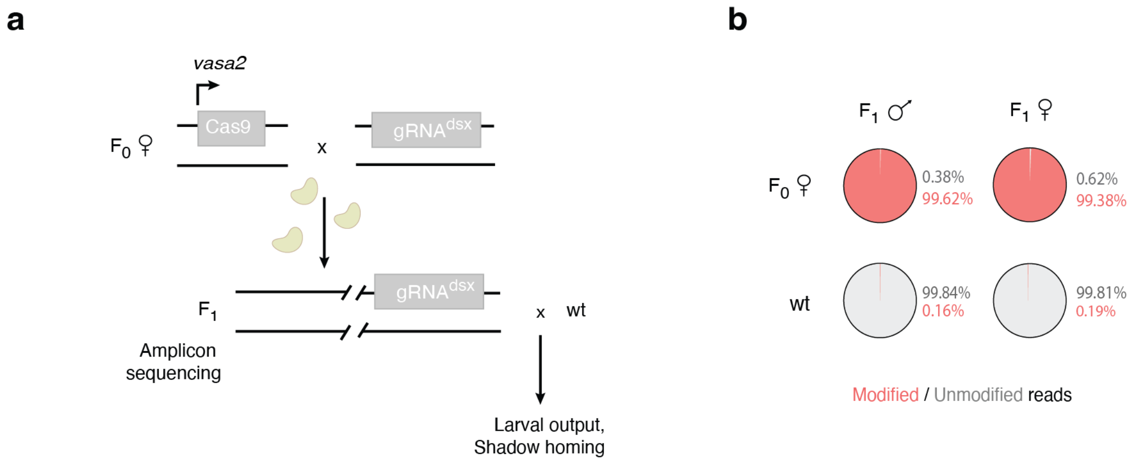

Supplementary Fig. 8. Assessment of parental Cas9 deposition induced by *vasa2* promoter. a Schematic of the genetic crosses performed to assess maternal Cas9 deposition induced by *vasa2* promoter. *Vasa2*:Cas9 females were crossed to gRNA^{dsx} males, and the F_1 progeny encoding only the gRNA^{dsx} (n=50) were isolated for pooled amplicon sequencing. **b** Percentage of unmodified (grey) vs modified (pink) reads from pooled amplicon sequencing at the *dsx* target site in the F_1 progeny inheriting only the gRNA^{dsx} (n=50).

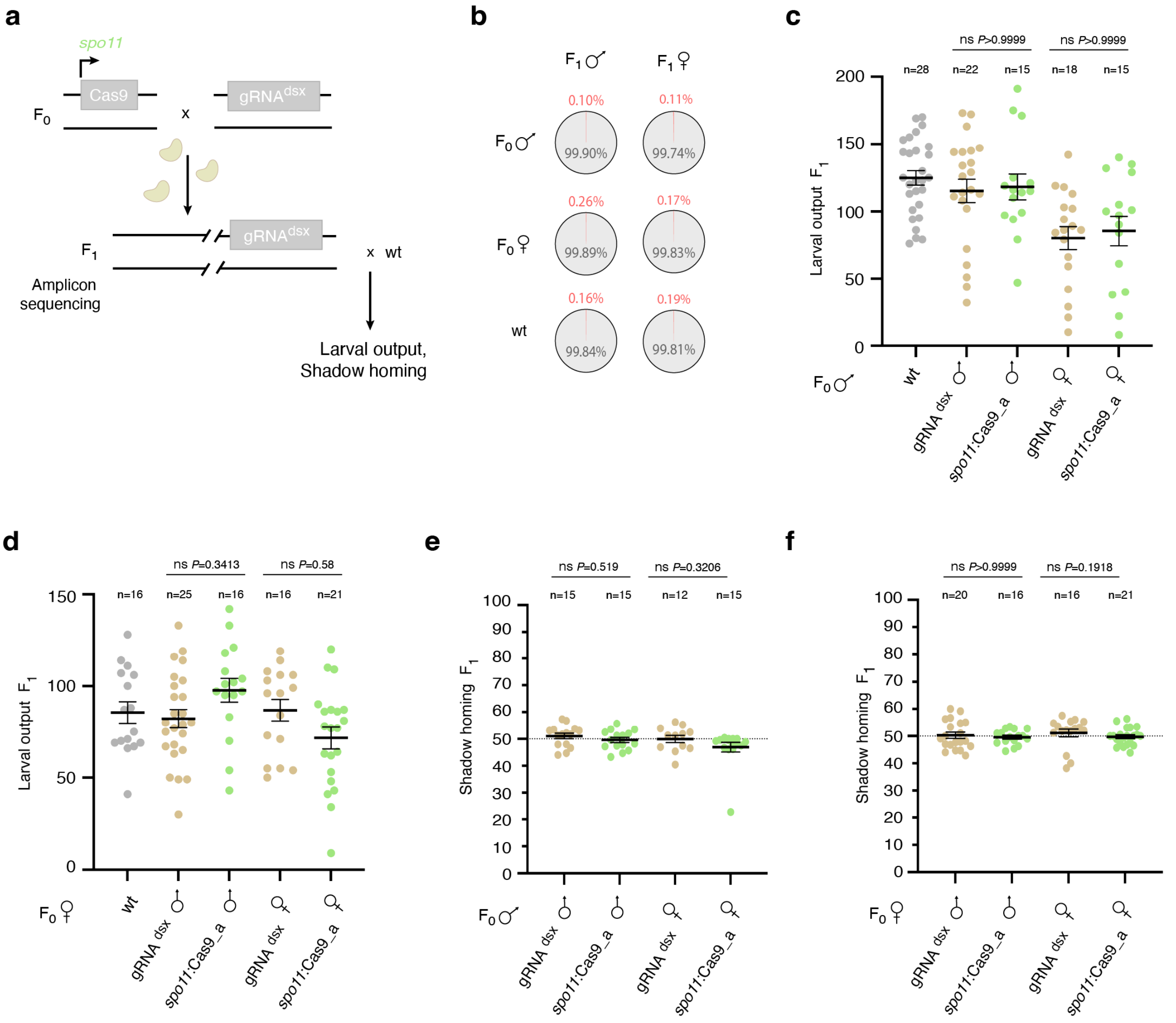

Supplementary Fig. 9. Assessment of parental Cas9 deposition induced by *spo11* promoter. a Schematic of the genetic crosses performed to test whether *spo11* promoter induces maternal and/or paternal deposition of the Cas9 endonuclease. The F_1 progeny encoding only the gRNA^{dsx}, and potentially bearing deposited Cas9, were selected for further analysis. **b** Percentage of unmodified vs modified reads from pooled amplicon sequencing (n=100) at the *dsx* target site in the F_1 progeny inheriting only the gRNA^{dsx}. F_1 gRNA^{dsx}-only males and females were outcrossed to their wild-type counterpart and females separated for egg-laying. **c-f** gRNA^{dsx}-only progeny of *spo11*:Cas9_a (green) was counted at egg and larval and scored for RFP inheritance to assess fecundity and shadow homing, respectively. Control groups include wild type

(grey) and the gRNA^{dsx} strain (beige). Dots represent the progeny of a single mosquito. The arithmetic mean of hatching and homing rates is indicated in percentages above each data set. Thick horizontal lines with error bars indicates the arithmetic mean and s.e.m. of larval output (**c,d**) and homing rates (**e,f**). Statistical analysis of fecundity of F1 vs gRNA^{dsx} line was performed using Kruskal-Wallis test followed by two-sided Dunn's multiple comparison test with the gRNA^{dsx} control. P values and sample size (n) are indicated in the figure above each data group. Source data are provided as a Source Data file. Graphs and statistical analysis were generated using GraphPad Prism (v10.6.0).

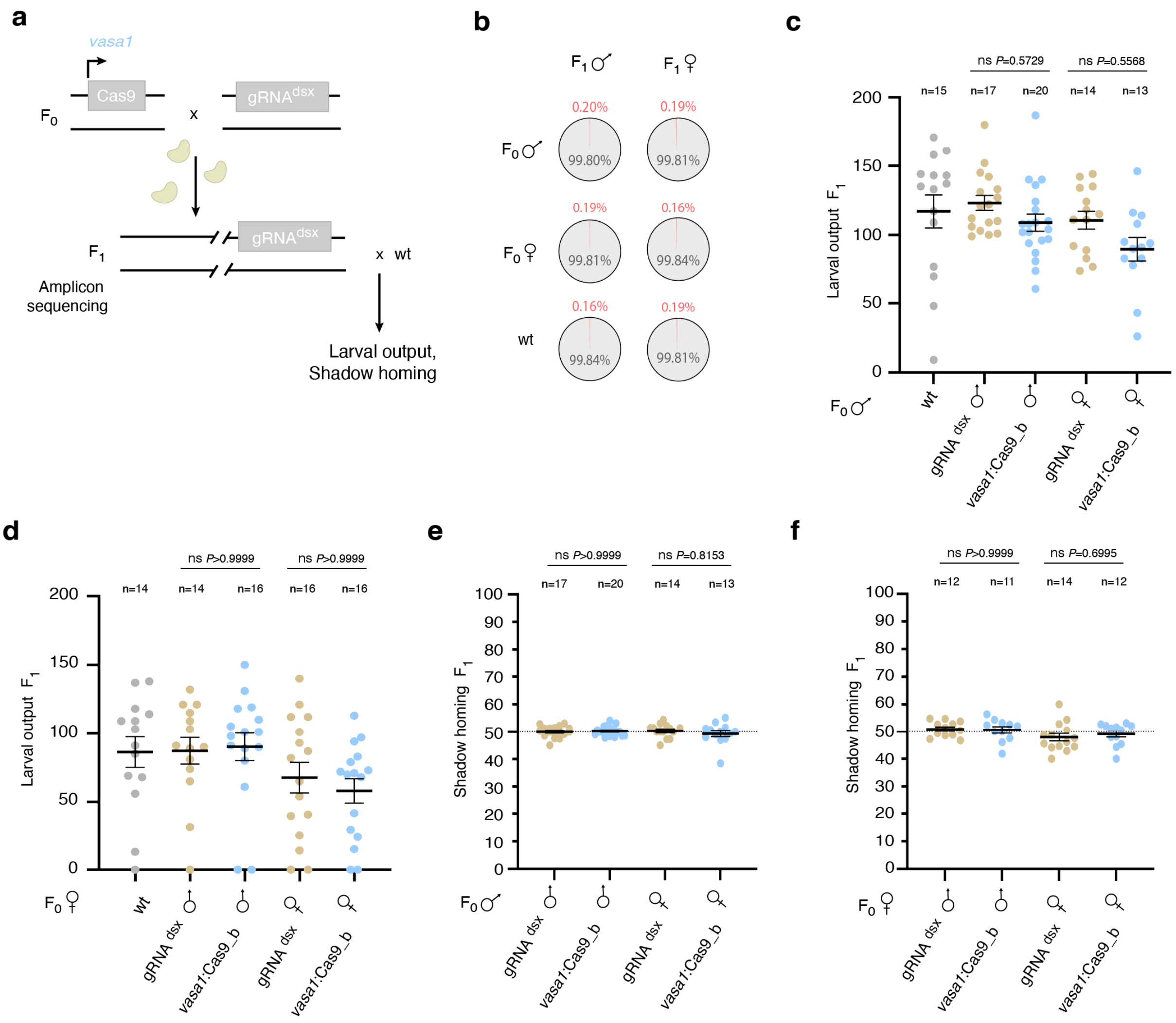

Supplementary Fig. 10. Assessment of parental Cas9 deposition induced by *vasa1* promoter. a Schematic of the genetic crosses to test whether *vasa1* promoter induces maternal and/or paternal deposition of Cas9 endonuclease. The F_1 progeny encoding the gRNA^{dsx}, and potentially bearing deposited Cas9, were selected for further analysis. **b** Percentage of unmodified vs modified reads from pooled amplicon sequencing (n=100) at the *dsx* target site in the F_1 progeny inheriting only the gRNA^{dsx}. F_1 gRNA^{dsx}-only males and females were outcrossed to their wild-type counterpart and females separated for egg-laying. **c-f** gRNA^{dsx}-only progeny of *vasa1*:Cas9_b strain (blue) was counted at egg and larval and scored for RFP inheritance to assess fecundity and shadow homing, respectively. Control groups include wild type (grey)

and the gRNA^{dsx} strain (beige). Dots represent the progeny of a single mosquito. Thick horizontal lines with error bars indicates the arithmetic mean and s.e.m. of larval output (**c,d**) and homing rates (**e,f**). Statistical analysis of fecundity of F1 vs gRNA^{dsx} line was performed using Kruskal-Wallis test followed by two-sided Dunn's multiple comparison test with the gRNA^{dsx} control. P values and sample size (n) are indicated in the figure above each data group. Source data are provided as a Source Data file. Graphs and statistical analysis were generated using GraphPad Prism (v10.6.0).

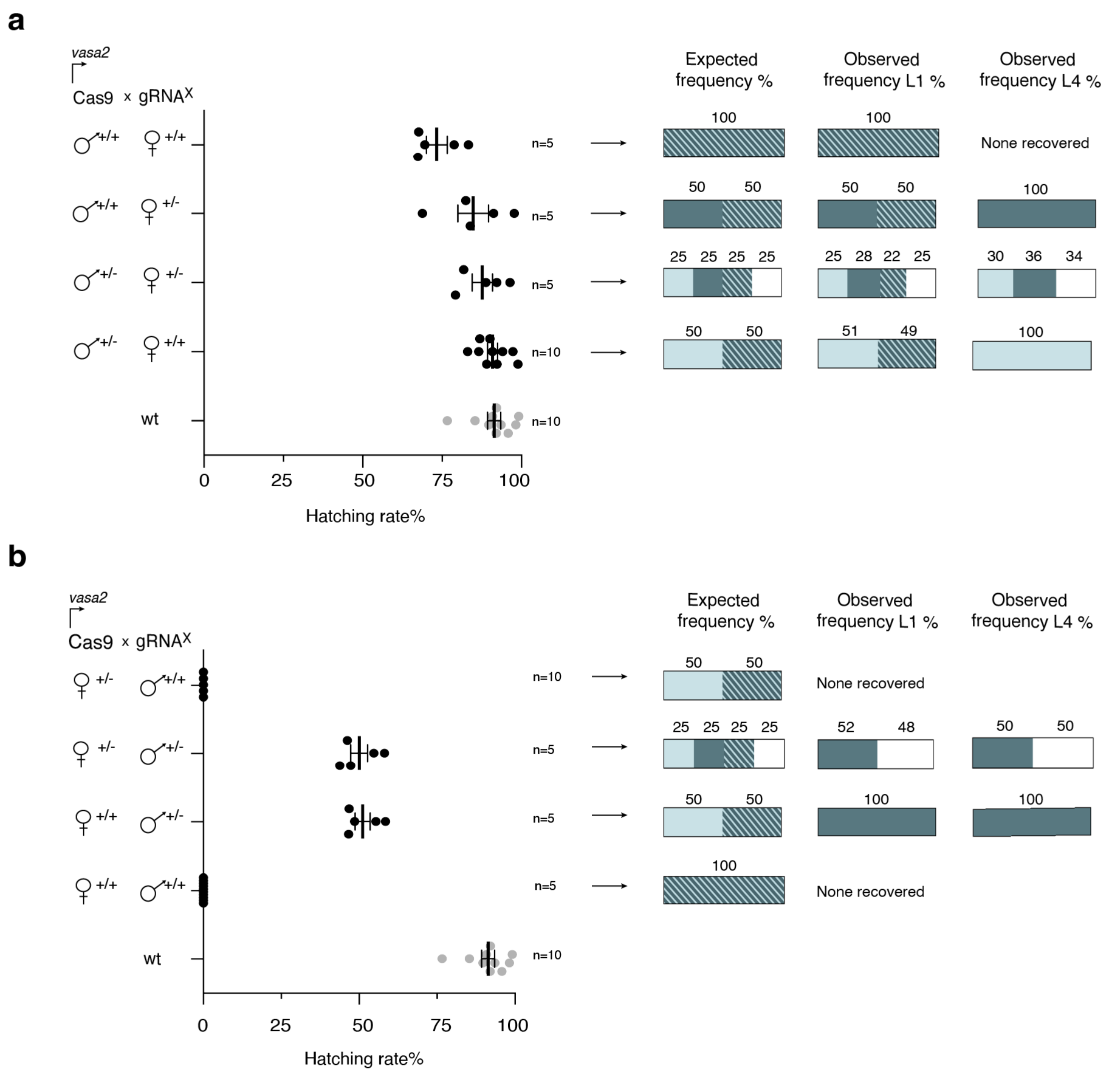

Supplementary Fig. 11 *vasa2*:Cas9 X-shredding. a *vasa2*:Cas9 males and (**b**) *vasa2*:Cas9 females (*3xP3*:GFP+) were crossed *en masse* to their $gRNA^X$ counterpart (*3xP3*:RFP+). Females were blood fed and separated individually for egg laying. Eggs and larvae were counted, and larvae were screened for both GFP and RFP marker to assess the frequency of each genotype, i.e. *vasa2*:Cas9 (teal), $gRNA^X$ (cyan) *vasa2*:Cas9/$gRNA^X$ (teal striped), and wild type (white), in comparison to the expected Mendelian frequency. When the Cas9 is inherited paternally (**a**), the trans-heterozygous *vasa2*:Cas9/$gRNA^X$ die at L2-L3 larval stages. When the Cas9 is inherited maternally (**b**), both the trans-heterozygous *vasa2*:Cas9/$gRNA^X$ and the $gRNA^X$ die at the embryonic stage. Dots represent the progeny of a single mosquito, and the sample size (n) is indicated in the figure next to each data group. Source data are provided as a Source Data file. Graphs were generated using GraphPad Prism (v10.6.0).

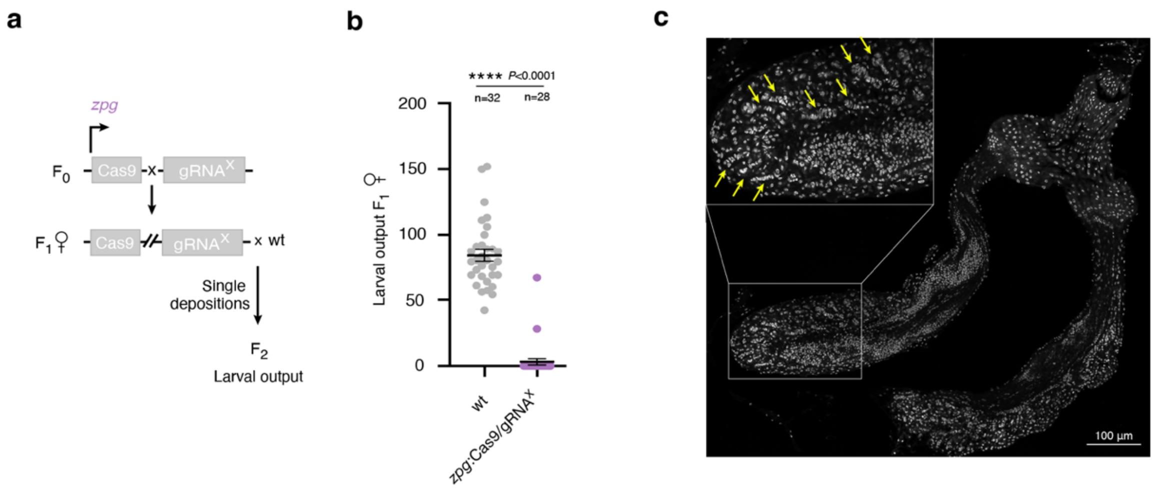

Supplementary Fig. 12 *zpg*:Cas9 X-shredding. a A previously generated strain harbouring a *zpg*:Cas9 transgene on chromosome 2L (20B) was crossed to gRNA^X. Trans-heterozygous *zpg*:Cas9/ gRNA^X females were crossed to wild type males and their fecundity (**b**) assessed by larvae counts (purple) in comparison to wild types (grey). Dots represent the progeny of a single female mosquito. Thick horizontal lines with error bars indicate the arithmetic mean and s.e.m. of larval output. Statistical analysis of larval output was performed using two-sided Mann Whitney test. P values and sample size (n) are indicated in the figure above each data group. **c** Confocal image of ovaries DAPI-stained *zpg*:Cas9/gRNA^X females, showing severe underdevelopment. The yellow arrows indicated stack of terminal filaments, which might be indicative of gametogenesis arrest at early stages. DAPI staining was performed on four samples. Scale bar=100 µm. Source data are provided as a Source Data file. Graphs and statistical analysis were generated using GraphPad Prism (v10.6.0).

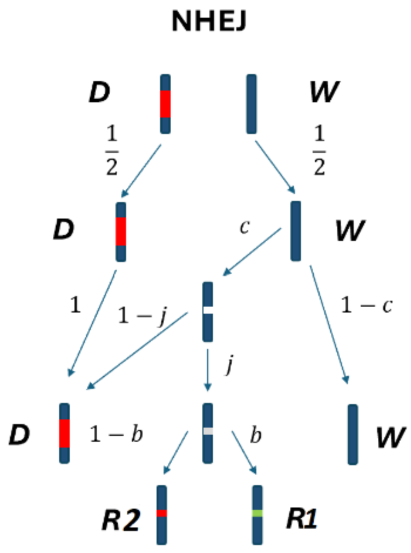

Supplementary Fig. 13. Diagram of gametogenesis for WD individuals (W= wild type, D= drive). The drive allele (D) is represented by a long red bar inserted at the target locus. Cleavage of the corresponding wild type allele is indicated by a short white bar. Repair of the cleaved allele via homologous recombination (HR) converts the wild type allele into a drive allele, resulting in super-Mendelian inheritance of the drive (D). Alternatively, repair through non-homologous end joining (NHEJ, short grey bar) can generate resistant alleles, either functional (R1, short green bar) or non-functional (R2, short red bar).

Strain	Fragment	BLAST hit	Cytogenic band	Score (bits)	E-value	Identities	Gaps
spo11:Cas9_a	5’	AgamP4_2L: 4448130-4459825 AGAP004850 (3’utr)	20D	152	3e-36	84/84 (100%)	0/84 (0%)
spo11:Cas9_b	5’	AgamP4_3L: 18439494-18439724	41C	408	5e-113	229/231 (99%)	0/231 (0%)
spo11:Cas9_c	3’	AgamP4_UNKN: 2154943-2155038	Exact insertion site cannot be uniquely determined due to weak hits	33.7	0.64	18/18 (100%)	0/18 (0%)
	3’	AgamP4_2R: 467473-467568		35.6	0.34	22/24 (92%)	0/24 (0%)
	5’	AgamP4_3R: 17133142-17135472		37.4	0.16	31/37 (84%)	1/37 (3%)
	5’	AgamP4_2L: 45612392-45633848		35.6	0.55	19/19 (100%)	0/19 (0%)
vasa1:Cas9_a	3’	AgamP4_3R: 28475762-28476020	33C	468	8e-131	259/259 (100%)	0/259 (0%)
vasa1:Cas9_b	3’	AgamP4_2R: 35259394-35259562	14D	277	1e-72	164/170 (96%)	1/170 (1%)
vasa1:Cas9_c	3’	AgamP4_3L: 11094643-11094738	40A	108	8e-23	67/72 (93%)	5/72 (7%)

Supplementary Table 1. Inverse PCR (iPCR) of *spo11*:Cas9 and *vasa1*:Cas9 strains. The table illustrates the top nBLAST hits of iPCR-fragment amplified from gDNA of *spo11*:Cas9 or *vasa1*:Cas9 strains, aligned against the *An. gambiae* PEST genome. For each strain, alignment scores, e-values, sequences identities and gaps are described, alongside the most likely corresponding genomic loci. The integration locus of *spo11*:Cas9_c strain remains elusive, as the two top hits for the 5’ and 3’ fragments are weak, showing only partial alignments, suggesting potential mapping to repetitive regions.

Strain	Cross		n	Cas9 + only (%)			gRNA + only (%)			Cas9+ / gRNA + (%)			Negatives (%)		
	Cas9	gRNA *dsx*		♀	♂	mosaics	♀	♂	mosaics	♀	♂	mosaics	♀	♂	mosaics
spo11:Cas9_a	♀	♂	207	12.1	13.0	0	13.5	12.6	0	8.7	10.1	0	15.9	14.0	0
	♂	♀	199	13.1	14.1	0	11.6	11.1	0	13.6	12.6	0	12.6	11.6	0
spo11:Cas9_b	♀	♂	228	13.6	14.5	0	12.7	11.8	0	14.5	13.2	0	11.0	8.8	0
	♂	♀	203	13.3	13.8	0	13.8	12.3	0	14.3	14.3	0	9.9	8.4	0
spo11:Cas9_c	♀	♂	171	8.8	11.1	0	11.7	12.9	0	14.6	14.0	0	15.2	11.7	0
	♂	♀	139	11.5	20.1	0	10.1	12.9	0	10.8	12.2	0	12.2	10.1	0
vasa1:Cas9_a	♀	♂	178	12.9	12.4	0	15.2	12.4	0	11.2	10.7	1.1	11.2	12.9	0
	♂	♀	135	17.0	15.5	0	8.9	10.4	0	10.4	14.1	0.7	10.4	12.6	0
vasa1:Cas9_b	♀	♂	300	12.3	13.0	0	13.0	14.3	0	10.0	12.0	1.0	11.7	12.7	0
	♂	♀	182	15.4	11.0	0	12.6	13.8	0	11.5	12.1	0.5	11.5	11.5	0
vasa1:Cas9_c	♀	♂	252	11.5	13.9	0	14.7	11.9	0	12.3	13.1	0.40	11.5	10.7	0
	♂	♀	289	12.5	17.3	0	9.3	14.9	0	12.1	12.1	1.0	9.7	11.1	0

Supplementary Table 2. Assessment of sexual mosaicism in the progenies of *spo11*:Cas9 or *vasa1*:Cas9 crosses with gRNA^{dsx} strain. The table shows the genotype composition of the offspring of *spo11*:Cas9 and *vasa1*:Cas9 strains crossed to the gRNA^{dsx} strain. The percentage of male and female offspring carrying either Cas9 or gRNA^{dsx}, both, or neither is illustrated. Presence of mosaic individuals in either Cas9-only or gRNA-only progeny reflects gRNA^{dsx} or Cas9 deposition, respectively. Presence of mosaic individuals in Cas9/gRNA^{dsx} individuals is indicative of somatic leakiness. Sample sizes (n) are indicated for each cross.

Target	Primer name	Sequence 5’-3’
***spo11* promoter**	*spo11*_P_F	TGATGGATGACGCCTTAAGAGAAAGATCAATAGCCAGCTAC
	*spo11*_P_R	CAGCTGTAGGCGCCGTGTGTTCCACGGTCG
***spo11* terminator**	*spo11*_T_F	GGACACTATCTGTCATCATGCGCAGAGTATTTAG
	*spo11*_T_R	TCCTCCTCCTGTGGGCATACCTACAGACAG
**iPCR**	5’_F1	GACGCATGATTATCTTTTACGTGAC
	5’_R1	TGACACTTACCGCATTGACA
	5’_F2	GCGATGACGAGCTTGTTGGTG
	5’_R2	TCCAAGCGGCGACTGAGATG
	3’_F1	CAACATGACTGTTTTTAAAGTACAAA
	3’_R1	GTCAGAAACAACTTTGGCACATAT
	3’_F2	CCTCGATATACAGACCGATAAAAC
	3’_R2	TGCATTTGCCTTTCGCCTTAT
	pB_5SEQ	CGCGCTATTTAGAAAGAGAGA
	pB_3SEQ	CGATAAAACACATGCGTCAATT
**SDMD integration**	*dsx*_e4_F1	CGACGAGCTAGTGAAGCGAGC
	gRNAX_R1	GGGTACAAGCTTGCGTACGT
	RFP_F2	GCGTGATGAACTTCGAGGAC
	*dsx*_e5_R2	TAACTGTCCGTATCTTTGTATGTGG
**Amplicon sequencing *dsx***	Illumina-AmpEZ-4050-F	ACACTCTTTCCCTACACGACGCTCTTCCGATCTACTTATCGGCATCAGTTGCG
	Illumina-AmpEZ-4050-R	GACTGGAGTTCAGACGTGTGCTCTTCCGATCTGTGAATTCCGTCAGCCAGCA
**Amplicon sequencing rDNA**	Illumina-AmpEZ-rDNA-F	ACACTCTTTCCCTACACGACGCTCTTCCGATCTGTAGCATCGTCGTGTGTAGC
	Illumina-AmpEZ-rDNA-R	GACTGGAGTTCAGACGTGTGCTCTTCCGATCTCCTTTGGACACCTCCGTTATC

Supplementary Table 3. List of primers. The table describes the sequences of the primers used to amplify the regulatory sequences of *spo11* (*spo11*_P_F/R), to characterise the genomic insertion site of *vasa1*:Cas9, *spo11*:Cas9 (iPCR primers)

and SDMD (*dsx*_e4_F1/ gRNAX_R1; RFP_F2/*dsx*_e5_R2) strains, and to analyse mutations at the dsx (Illumina-AmpEZ-4050-F/R) and rDNA (Illumina-AmpEZ-rDNA-F/R) loci through amplicon sequencing. Illumina partial adapter sequences in amplicon sequencing primers are underlined.

Strain	Injected embryos	Transient males (G_0)	Transient females (G_0)	Non-transient males (G_0)	Non-transient females (G_0)	Transgenic males (G_1)	Transgenic females (G_1)
***spo11*:Cas9**	532	6	8	33	28	16	5
***vasa1*:Cas9**	502	10	6	40	42	8	2
**SDMD**	2520	42	40	254	270	1	1

Supplementary Table 4. Embryo microinjections of genetic constructs to develop the *spo11*:Cas9, *vasa1*:Cas9 and SDMD strains. For each strain, the number of embryos injected and G_0 larvae recovered, classified as “transient” and “non transient” accordingly to fluorescent marker expression is illustrated. G_1 transgenic individuals in the progeny of G_0 individuals outcrossed to wild types were selected to establish the corresponding transgenic strain.

Genotype	Gametes produced				Fitness	
	W	D	R2	R1	Females	Males
**WW**	1	0	0	0	1	1
**WD**	$\frac{(1-c)}{2}$	$\frac{c(1-j)}{2}$	$\frac{cj(1-b)}{2}$	$\frac{cjb}{2}$	$1-h_D s$	1
**WR2**	½	0	½	0	$1-h_{R2} s$	1
**WR1**	½	0	0	½	1	1
**DD**	0	1	0	1	$1-s$	1
**DR2**	0	½	½	0	$1-s$	1
**DR1**	0	½	0	½	$1-h_{D,R1} s$	1
**R2R2**	0	0	1	0	$1-s$	1
**R2R1**	0	0	½	½	$1-h_{R2} s$	1
**R1R1**	0	0	0	1	1	1

Supplementary Table 5 Gametes production probabilities and fitness parameters of SDMD. The genotypes represented in the table refer to the following alleles: W= wild type, D= drive, R1= functional resistant, R2= non-functional resistant. Fitness costs are female-specific; fitness is relative to wild-type.

Genotype	Female fitness		
	GD	SDGD	SDMD
**WW**	1	1	1
**WD**	1	1	0
**WR2**	1	1	1
**WR1**	1	1	1
**DD**	0	0	0
**DR2**	0	0	0
**DR1**	1	1	0
**R2R2**	0	0	0
**R2R1**	1	1	1
**R1R1**	1	1	1
**Sex ratio**	0.5	0.95	0.95
**Male fitness**	1	1	1

Supplementary Table 6 Parameter values for the generalised modelling comparisons of gene drive (GD), sex distorter gene drive (SDGD) and sex distorter male drive (SDMD) strategies. The table describes the parameters used to model the idealised GD, SDGD and SDMD strategies shown in Figure 1. The genotypes represented in the table correspond to the following alleles: W= wild type, D= drive, R1= functional resistant, R2= non-functional resistant. Fitness costs are female-specific, with only full sterility or full fertility considered.

Parameter	Description	Baseline value		
		GD	SDGD	SDMD
N	Initial population size		10^15	
f	Average number of eggs (Rm=6)		12	
c	Cleavage rate (GD, SDGD)	0.966	0.9576	0.7556
j	Non-homologous end-joining frequency given cleavage	0.035	0.0392	0.0206
b	Probability NHEJ product is functional (R1)		0.0016	
m	Sex distortion ratio of the offsprings of males with D allele	0.5	0.93	0.914
s	Fitness cost of nonfunctional homozygotes (D or R2)	1	1	1
$h_{D,R1}$	Fitness cost dominance coefficient for DR1 females	0	0	1
h_D	Fitness cost dominance coefficient for WD females	0	0	1
h_{R2}	Fitness cost dominance coefficient for R2 allele (WR2, R1R2 females)	0	0	0

Supplementary Table 7. Parameter values used for modelling specific gene drive (GD), sex distorter gene drive (SDGD) and sex distorter male drive (SDMD) targeting *dsx* based on empirical data. The table describes the parameters used to model the GD, SDGD and SDMD strategies shown in Figure 6. Parameters for the GD and SDGD strategies are derived from previously developed systems targeting the *dsx* locus[3,4]. Parameters for the SDMD are derived from this study. Alleles are denoted as follows: W = wild type, D = drive, R1= functional resistant, R2= non-functional resistant.

References

(1) Guichard, A.; Haque, T.; Bobik, M.; Xu X.; Klanseck, C.; Kushwah, R.B.; Berni, M.; Kaduskar, B.; Gantz, V.M. and Bier, E. Efficient allelic-drive in *Drosophila*. *Nat Commun* **2019,** 10(1640). https://doi.org/10.1038/s41467-019-09694-w.

(2) Hammond, A. M.; Kyrou, K.; Bruttini, M.; North, A.; Galizi, R.; Karlsson, X.; Kranic, N.; Carpi, F. M.; D'Aurizio, R.; Crisanti, A.; Nolan, T. The Creation and Selection of Mutations Resistant to a Gene Drive over Multiple Generations in the Malaria Mosquito. *PLoS Genet* **2017**, *13* (10). https://doi.org/10.1371/journal.pgen.1007039.

(3) Morianou, I.; Phillimore, L.; Khatri, B. S.; Marston, L.; Gribble, M.; Burt, A.; Bernardini, F.; Hammond, A. M.; Nolan, T.; Crisanti, A. Engineering Resilient Gene Drives Towards Sustainable Malaria Control: Predicting, Testing and Overcoming Target Site Resistance. bioRxiv October 21, 2024, p 2024.10.21.618489. https://doi.org/10.1101/2024.10.21.618489.

(4) Simoni, A.; Hammond, A. M.; Beaghton, A. K.; Galizi, R.; Taxiarchi, C.; Kyrou, K.; Meacci, D.; Gribble, M.; Morselli, G.; Burt, A.; Nolan, T.; Crisanti, A. A Male-Biased Sex-Distorter Gene Drive for the Human Malaria Vector Anopheles Gambiae. *Nat Biotechnol* **2020**, *38* (9), 1054–1060. https://doi.org/10.1038/s41587-020-0508-1.
