# Supplementary material for: Sex distorter male drive for resistance-resilient population control of the human malaria vector Anopheles gambiae
Source: Nat Commun. 2026 Apr 11;17:5109. doi: 10.1038/s41467-026-71627-1 (PMC13246785; doi:10.1038/s41467-026-71627-1)
Supplement: Supplementary file 3 — Transparent Peer Review file [file 41467_2026_71627_MOESM3_ESM.pdf]

nature portfolio

Peer Review File

Sex Distorter Male Drive for resistance-resilient population control of the human malaria vector Anopheles gambiae

Corresponding Author: Dr Federica Bernardini

This file contains all reviewer reports in order by version, followed by all author rebuttals in order by version.

Version 0:

Reviewer comments:

Reviewer #1

(Remarks to the Author)
The manuscript ebtitled “Sex Distorter Male Drive for efficient, resistance-resilient population control of the human malaria vector Anopheles gambiae” is well written and contributes to an important area of vector biology, specifically the development of genetic tools to bias sex ratios and enhance the efficiency of gene drive systems in Anopheles gambiae. The identification and characterization of two germline-specific promoters (spo11 and vasa1) with distinct temporal and spatial expression patterns offer valuable insights and resources for the field. The subsequent application of these promoters to engineer a Sex Distorter Male Drive that produces a strongly male-biased progeny represents a promising advance towards Gene Drive technology for vector control.
Our main concern is that there are no cage or semi-field cage results to confirm the gain of efficiency and the claimed gain against resistance development, which are only evaluated based on models. Also, we have some concerns that should be addressed before it can be considered for publication.
Major concerns
- L319-320 and M&M section: the fitness of the transgenic strains was evaluated only through larval output and survival. However, a strain can reproduce and survive in lab conditions but have very poor fitness after release: it would have been useful to include competitiveness and flight ability tests, as described in 1 for example.
- In fig. 2g, the larval throughputs of the control groups (wt) are completely different between the experiments evaluating vasa 1 and spo 11 male F1: why this? Normally, they should be identical. Same in fig 3e.
- In fig 6, the integrated suppression area is higher for SDMD than GD an SDGD, but the initial suppression rate is much lower. Why is this? Even if the escaped females are sterile with SDGD, won’t it still be more likely to observe resistance if the residual population is much bigger (eg ~25% of the initial population instead of almost 0% with GD & SDGD)? If the mosquito population is reduced by 75%, will this reduce malaria incidence? We did not see this result presented nor discussed anywhere in the paper.

Minor concerns
- In the figure legends, it would be necessary to explain the abbreviations so that the figures can be stand-alone
- L196 : is “£1%” equivalent to “~1%”?
- L519 Mosquito rearing : The manuscript would benefit from a clearer description of the mosquito strain maintained in the laboratory. Please specify the strain name, its geographic origin, and any relevant characteristics. This information can be essential for reproducibility and for contextualizing the experimental results, especially when comparing field and laboratory populations.
- L645 Pooled amplicon sequencing: Please indicate the number of mosquitoes pooled per DNA extraction and provide a brief rationale for the pooling strategy. This information could be essential for assessing the reproducibility, the representativeness of the samples and the potential impact on downstream analyses.
- L571-573 : “All G0 survivors were separated according to the presence or absence of transient expression of the fluorescent marker in the terminal part at L1 larval stage and crossed to wild types.” Please specify the age of mosquitoes when the crossing was performed.
- L579-582 : “To characterize the spo11:Cas9 and vasa1:Cas9 strains, inverse PCR was performed as previously described to characterize the genomic location of the transgene, using iPCR primers shown in Supplementary Table S4." It would be interesting to show or give more details on the minimum size of fragment used for iPCR since the accuracy may depend on it.
- L665 : "They assessed Cas9 deposition." It would also be interesting to assess the leakiness of these regulatory elements.

The fact that there is no Cas9 deposition does not necessarily imply that there is no leaky expression. and if there is a leaky expression, we will still face the same issue.
- L700-701 Mathematical modeling: The mention of code availability is repeated twice with similar wording and the same GitHub link. Please place it in the section 'code availability'.

Reference cited
1 Lutrat, C. et al. Combining two Genetic Sexing Strains allows sorting of non-transgenic males for Aedes genetic control. Commun. Biol 6, 646, doi:10.1038/s42003-023-05030-7 (2023).

(Remarks on code availability)

Reviewer #2

(Remarks to the Author)
Grilli et al. in their manuscript "Sex Distorter Male Drive for efficient, resistance-resilient population control of the human malaria vector Anopheles gambiae" describe a promising male-biased homing gene drive system and characterise two germline promoters which can simplify the genetic constructs used to develop such systems. Single germline Cas9 for both homing at a female fertility gene loci and X-Shredding is a novel strategy. The research is technically sound, and the article is well-structured with appropriate subsections, making it easy to follow and enjoyable to read.

However, I believe the following comments should be addressed to provide more information for readers and to enhance the overall quality of the manuscript. Apart from that, I think this article aligns well with the scope of Nature Communications and would be a valuable addition to the development of gene drive systems aimed at controlling mosquito vectors.

Major Comments:

Regarding the characterisation of vasa1 and spo11 germline promoters and understanding maternal deposition, I wonder if the authors examined the zygotic expression pattern of Cas9 transcripts driven by vasa1 and spo11 over a time course (since no such data appear to be presented). Specifically, post-blood meal (maternal) and post-oviposition (zygotic). This would provide a clearer understanding of the expression profiles of these regulatory promoter regions. Additionally, replicating this across different transgenic lines would help to understand positional effects on germline gene expression. Although this may be outside the scope of this article, bioinformatic analyses of the promoter regions could identify regulatory elements / TF binding sites. This could help in selecting key regions and even allow a reduction in the length of the promoter elements, thereby decreasing the size of the genetic constructs.

Regarding the fitness costs of the initial transgenic lines, the authors have only performed fertility assays, examining hatching rates and larval counts. If data on pupation rates, pupal eclosion or emergence are available, including these would help to understand any other fitness costs associated with these lines.

Also, if the authors have records of the injection experiments performed to develop different Cas9 and SDMD lines, sharing this information would benefit others aiming to develop similar systems.

Minor Comments:

Throughout the article, the use of British or American spelling (characterise/characterize, characterised/characterized) was used, and the authors can be consistent.

Microscopy images in Figures 3F & 4D were quite small. Enlarging these would improve clarity. For better utilisation of space, Figures 3D/3G could be combined like Figure 3A.

Line 658/659: "DAPI staining of reproductive organs, 3-day-old mosquito testes or 2-day-old unfed mosquito ovaries were dissected", I think the data is missing here, or no data is shown.

Line 196: "<" instead of "£"?

Also, in the figure captions for Figures 2 and 3, "£" symbols were used for the p-values. I think that's a typo.

Regarding data availability, the authors could also consider providing plasmid sequences. Only the primers used for cloning were listed in the supplementary file – either through a repository or as a GenBank file or similar.

PS: I really like the Figure 2B schematics of different insertion sites in one figure. Well done!

(Remarks on code availability)
Yes, I did check the github code using the above link. The code has a README file and additional information regarding the different packages and dependencies required to run the code. I didn't tried installing / running the code.

Reviewer #3

(Remarks to the Author)
I co-reviewed this manuscript with one of the reviewers who provided the listed reports. This is part of the Nature Communications initiative to facilitate training in peer review and to provide appropriate recognition for Early Career Researchers who co-review manuscripts.

(Remarks on code availability)
The code appears to be well structured, but I was unable to test its reproducibility.

Version 1:

Reviewer comments:

Reviewer #1

(Remarks to the Author)
I would like to thank the authors for their replies.

However, I am afraid I did not find in the manuscript the text corresponding to these replies in the rebuttal letter:
- "As discussed in the manuscript, we aim to optimise it to further mitigate the potential emergence of functional resistance, for instance by incorporating additional gRNAs. For these reasons, we believe that the extensive and time-consuming nature of cage trials looking at fitness effects would be more appropriately reserved for an optimised version of our construct. We have now added a paragraph to the discussion outlining this reasoning (L562-576)."
In the version of the manuscript I received (natcom_629129_1_uploaded_1762980501.pdf), I did not find such a paragraph L562-576, nor elsewhere.
- "The observed differences in wild-type larval throughputs likely reflect natural biological variation between the experiments, which were conducted separately. Various factors may have influenced these results, including differences in blood batches and rearing conditions, such as fluctuations in humidity or temperature. For this reason, and to ensure a valid comparison, each experiment included its own wild-type control, treated identically to the experimental group."
I am sorry but strong variations between control groups but also within control groups (from 0 to 120 larvae per female in fig 4f) also reflect poor ability of the team to control heterogeneity (like the quality of the blood…). Using absence of differences between strains to conclude that fitness are the same is then quite risky. This should be acknowledged as a limitation.
- "As for the effect of a quantitative reduction (75%) in mosquito population size on malaria incidence, this will depend on the circumstances, but vectorial capacity and the basic reproduction number of the malaria are expected to be linear functions of the number of female mosquitoes. We have now added wording on this point (L572-576)."
Sorry but I could not find this added wording L572-576 or elsewhere in the manuscript. Also, as a specialist of vector-borne disease transmission: no, malaria transmission does not decrease linearly with vector density.

Finally, I don't see the point in publishing improved gene drive systems without clearly linking the results to the expected outputs, ie vector suppression else than from model predictions and more importantly malaria incidence. Here you show a better resilience to resistance, but not efficiency, contrary to what is stated in the title.

(Remarks on code availability)

Reviewer #2

(Remarks to the Author)
The authors have addressed all my comments and revised the manuscript. I am satisfied with the changes, and I believe the quality has improved.

(Remarks on code availability)

Reviewer #3

(Remarks to the Author)
I co-reviewed this manuscript with one of the reviewers who provided the listed reports. This is part of the Nature Communications initiative to facilitate training in peer review and to provide appropriate recognition for Early Career Researchers who co-review manuscripts.

(Remarks on code availability)
Yes, the code is reproducible, usable, and includes clear instructions for installation and execution.

Version 2:

Reviewer comments:

Reviewer #1

(Remarks to the Author)
We are satisfied with the new version of the manuscript. Thanks for your efforts.

(Remarks on code availability)

Reviewer #3

(Remarks to the Author)
I co-reviewed this manuscript with one of the reviewers who provided the listed reports. This is part of the Nature Communications initiative to facilitate training in peer review and to provide appropriate recognition for Early Career Researchers who co-review manuscripts.

(Remarks on code availability)
The code is fully reproducible, well documented, and constitutes an immediately usable resource for the community.

Open Access This Peer Review File is licensed under a Creative Commons Attribution 4.0 International License, which permits use, sharing, adaptation, distribution and reproduction in any medium or format, as long as you give appropriate credit to the original author(s) and the source, provide a link to the Creative Commons license, and indicate if changes were made.
In cases where reviewers are anonymous, credit should be given to 'Anonymous Referee' and the source.
The images or other third party material in this Peer Review File are included in the article’s Creative Commons license, unless indicated otherwise in a credit line to the material. If material is not included in the article’s Creative Commons license and your intended use is not permitted by statutory regulation or exceeds the permitted use, you will need to obtain permission directly from the copyright holder.
To view a copy of this license, visit https://creativecommons.org/licenses/by/4.0/

REVIEWER COMMENTS

Reviewer #1 (Remarks to the Author):

The manuscript entitled “Sex Distorter Male Drive for efficient, resistance-resilient population control of the human malaria vector Anopheles gambiae” is well written and contributes to an important area of vector biology, specifically the development of genetic tools to bias sex ratios and enhance the efficiency of gene drive systems in Anopheles gambiae. The identification and characterization of two germline-specific promoters (spo11 and vasa1) with distinct temporal and spatial expression patterns offer valuable insights and resources for the field. The subsequent application of these promoters to engineer a Sex Distorter Male Drive that produces a strongly male-biased progeny represents a promising advance towards Gene Drive technology for vector control.

1. Our main concern is that there are no cage or semi-field cage results to confirm the gain of efficiency and the claimed gain against resistance development, which are only evaluated based on models.

We thank the reviewer for their thoughtful comment regarding the inclusion of cage trial experiments. In our previous cage trials of population suppression gene drives, we observed the emergence of resistant alleles only in drives targeting the female fertility genes *AGAP007280*, *AGAP005958*, and *AGAP011377* (Hammond *et al*., 2016; Hammond *et al*., 2017; Hammond *et al*., 2021). The formation of these resistant alleles was primarily attributed to end-joining mutations resulting from maternally deposited nuclease activity in embryos and from unrestricted endonuclease expression outside the mosquito germline.
Subsequently, we refined our gene drive designs using two key strategies to mitigate the evolution of resistance: first, by restricting Cas9 expression to the germline through replacement of the *vasa2* promoter with the germline-specific *zpg* promoter; and second, by targeting a functionally constrained and highly conserved sequence within the *doublesex* gene. This optimised design resulted in complete population collapse in caged mosquito trials, without the emergence of functional resistance alleles (Kyrou *et al*., 2018).

To further enhance suppression efficiency, a sex-distorter element was incorporated into the gene drive to bias the progeny sex ratio toward males, thereby reducing opportunities for functional resistance to evolve, as such mutations are not selected in males (Simoni *et al*., 2020). The resulting sex-distorter gene drive (SDGD), integrated into the *dsx* locus, effectively collapsed caged mosquito populations in accordance with modelling predictions and showed no evidence of resistance (Simoni *et al*., 2020). In summary, no functional resistance has been detected in any previous cage trials involving *dsx*-targeting gene drives, either with or without the incorporated sex-distorter element, across both small- and large-scale experiments (Kyrou *et al*., 2018; Simoni *et al*., 2020; Hammond *et al*., 2021; D'Amato *et al*., 2024; Morianou *et al*., 2025).

In the present study, we developed a new strain, the sex-distorter male drive (SDMD), which achieves outcomes comparable to the previously tested SDGD technology, namely, super-Mendelian inheritance of the construct and a strongly male-biased progeny. The key distinction between the two strains lies in the phenotype of the escapee females, which remain fertile in the SDGD but are sterile in the SDMD. Mathematical modelling indicates that the dominant sterility imposed on 'escapee' females in the SDMD is expected to further delay the emergence of resistance compared to the SDGD, thereby achieving a more durable and sustained suppression of the target population.

This improvement is important because not observing resistance in the lab does not mean it will not be seen after a field release, and further efforts to prevent or delay it are important. The probability that resistance evolves before population elimination depends on the population size (Khatri & Burt 2019), and population sizes in the lab are necessarily much smaller than those in the field. Thus, modelling must play a more central role in our assessments against the evolution of resistance, while the primary role for cage experiments is to test for unexpected fitness effects.

However, the genetic construct described here represents a proof-of-principle design. As discussed in the manuscript, we aim to optimise it to further mitigate the potential emergence of functional resistance, for instance by incorporating additional gRNAs. For these reasons, we believe that the extensive and time-consuming nature of cage trials looking at fitness effects would be more appropriately reserved for an optimised version of our construct.

We have now added a paragraph to the discussion outlining this reasoning (L562-576).

Also, we have some concerns that should be addressed before it can be considered for publication.

Major concerns

2. L319-320 and M&M section: the fitness of the transgenic strains was evaluated only through larval output and survival. However, a strain can reproduce and survive in lab conditions but have very poor fitness after release: it would have been useful to include competitiveness and flight ability tests, as described in 1 for example.

We thank the reviewer for this insightful recommendation. We have now included a mating competitiveness experiment to better assess the fitness of SDMD heterozygous males. The results, presented in Figure 4j and described in L373–387 of the revised manuscript, show that SDMD males performed slightly below wild-type males in competition experiments; however, this difference was not statistically significant.

Regarding flight ability, we do not currently have access to a flight-testing device. Nonetheless, such experiments could be pursued in future studies with improved SDMD strains, potentially in collaboration with laboratories equipped with the necessary facilities to assess this phenotype. A line in the discussion was added to highlight the need for more comprehensive assessments of potential fitness effect on these transgenic males (L603-605)

3. In fig. 2g, the larval throughputs of the control groups (wt) are completely different between the experiments evaluating vasa 1 and spo 11 male F1: why this? Normally, they should be identical. Same in fig 3e.

We thank the reviewer for pointing this out, as the experimental setup might indeed require clarification. The observed differences in wild-type larval throughputs likely reflect natural biological variation between the experiments, which were conducted separately. Various factors may have influenced these results, including differences in blood batches and rearing conditions, such as fluctuations in humidity or temperature. For this reason, and to ensure a valid comparison, each experiment included its own wild-type control, treated identically to the experimental group.

We have now clarified in the corresponding figure legends (Figures 2 and 3) that these experiments were conducted independently. The figures were grouped in the manuscript to optimize space.

4. In fig 6, the integrated suppression area is higher for SDMD than GD an SDGD, but the initial suppression rate is much lower. Why is this? Even if the escaped females are sterile with SDGD, won't it still be more likely to observe resistance if the residual population is much bigger (eg ~25% of the initial population instead of almost 0% with GD & SDGD)? If the mosquito population is reduced by 75%, will this reduce malaria incidence? We did not see this result presented nor discussed anywhere in the paper.

Yes, the population size for SDMD does not decrease as far as for the other strategies; instead, the difference is in the much slower rate of recovery. This indicates that the difference is not in the rate of origin or initial establishment of the resistant allele (note these simulations are done with effectively infinite population size), but instead in the strength of selection for resistance once it has arisen. As for the effect of a quantitative reduction (75%) in mosquito population size on malaria incidence, this will depend on the circumstances, but vectorial capacity and the basic reproduction number of the malaria are expected to be linear functions of the number of female mosquitoes. We have now added wording on this point (L572-576).

Minor concerns

5. In the figure legends, it would be necessary to explain the abbreviations so that the figures can be alone

We have updated the figure legends to include explanations of all abbreviations.

6. L196 : is “£1%” equivalent to “~1%”?

This was a typographical error, which has now been corrected. “£1%” has been replaced with “<1%.”

7. L519 Mosquito rearing: The manuscript would benefit from a clearer description of the mosquito strain maintained in the laboratory. Please specify the strain name, its geographic origin, and any relevant characteristics. This information can be essential for reproducibility and for contextualizing the experimental results, especially when comparing field and laboratory populations.

We have updated the Materials and Methods section (L596–598) to provide a clearer description of the mosquito strain maintained in the laboratory, including its strain name (*An. gambiae* G3 strain) and geographic origin (Gambia, 1975).

8. L645 Pooled amplicon sequencing: Please indicate the number of mosquitoes pooled per DNA extraction and provide a brief rationale for the pooling strategy. This information could be essential for assessing the reproducibility, the representativeness of the samples and the potential impact on downstream analyses.

We have now indicated the number of individuals used for each pooling strategy in both the figure legends and the main text. Pooling multiple individuals per DNA extraction was employed to increase the number of samples analysed, ensuring their representativeness.

9. L571-573 : “All G0 survivors were separated according to the presence or absence of transient expression of the fluorescent marker in the terminal part at L1 larval stage and crossed to wild types.” Please specify the age of mosquitoes when the crossing was performed.

The cross was performed at the adult stage, three days after pupal eclosion, and blood feeding was provided after five days of mating. We have now included this information in the revised manuscript (L680-682).

10. L579-582 : "To characterize the spo11:Cas9 and vasa1:Cas9 strains, inverse PCR was performed as previously described to characterize the genomic location of the transgene, using iPCR primers shown in Supplementary Table S4." It would be interesting to show or give more details on the minimum size of fragment used for iPCR since the accuracy may depend on it.

We thank the reviewers for the suggestion. We have now reported such information in Supplementary Table S1.

11. L665 : "They assessed Cas9 deposition." It would also be interesting to assess the leakiness of these regulatory elements. The fact that there is no Cas9 deposition does not necessarily imply that there is no leaky expression. and if there is a leaky expression, we will still face the same issue.

We have previously shown that knockout of dsxF at this target site results in homozygous females exhibiting an intersex phenotype and full sterility, while heterozygous females remain fully fertile and phenotypically normal (Kyrou et al., 2018). When a Cas9 endonuclease is expressed from this locus under the control of the *zpg* promoter, leaky somatic expression can lead to mutagenesis in non-germline cells and unintended fitness costs (Kyrou *et al*., 2018).
In our study, we also observed leakiness of the *vasa1* regulatory element. In trans-heterozygous *vasa1*:Cas9/gRNA^{dsx} females, approximately 1% of individuals exhibited sexual mosaicism at the pupal stage, accompanied by mutations at the target site (Figure S3). Phenotypic assays further revealed fitness costs in trans-heterozygous *vasa1*:Cas9/gRNA^{dsx} adult females (Figure 2h). Conversely, no sexual mosaicism or fitness cost was observed in *spo11*:Cas9/gRNA^{dsx} individuals, suggesting that the *spo11* regulatory sequences do not exhibit leaky expression.

While promoter leakiness is typically considered a limitation in standard gene drive designs, as unintended somatic expression can reduce drive efficiency in

heterozygous individuals, in the strategy described here we deliberately leveraged the leakiness of *vasa1* to impose dominant sterility on SDMD females.

12. L700-701 Mathematical modeling: The mention of code availability is repeated twice with similar wording and the same GitHub link. Please place it in the section ‘code availability’.

The repeated mention of code availability has been removed, and the GitHub link is now included only in the ‘Code Availability’ section, as suggested.

Reference cited

1 Lutrat, C. et al. Combining two Genetic Sexing Strains allows sorting of non-transgenic males for Aedes genetic control. Commun. Biol 6, 646, doi:10.1038/s42003-023-05030-7 (2023).

Reviewer #2 (Remarks to the Author):

Grilli et al. in their manuscript “Sex Distorter Male Drive for efficient, resistance-resilient population control of the human malaria vector Anopheles gambiae” describe a promising male-biased homing gene drive system and characterise two germline promoters which can simplify the genetic constructs used to develop such systems. Single germline Cas9 for both homing at a female fertility gene loci and X-Shredding is a novel strategy. The research is technically sound, and the article is well-structured with appropriate subsections, making it easy to follow and enjoyable to read.

However, I believe the following comments should be addressed to provide more information for readers and to enhance the overall quality of the manuscript. Apart from that, I think this article aligns well with the scope of Nature Communications and would be a valuable addition to the development of gene drive systems aimed at

controlling mosquito vectors.

Major Comments:

13. Regarding the characterisation of vasa1 and spo11 germline promoters and understanding maternal deposition, I wonder if the authors examined the zygotic expression pattern of Cas9 transcripts driven by vasa1 and spo11 over a time course (since no such data appear to be presented). Specifically, post-blood meal (maternal) and post-oviposition (zygotic). This would provide a clearer understanding of the expression profiles of these regulatory promoter regions. Additionally, replicating this across different transgenic lines would help to understand positional effects on germline gene expression.

We thank the reviewer for this valuable suggestion. We did not perform transcript-level analyses because the availability of the transgenic strains allowed us to directly assess parental deposition (maternal and paternal) by measuring functional Cas9 protein activity in embryos, following approaches previously applied to the *vasa2*, *zpg*, and *nos* promoters (Hammond *et al*., 2021).

To preliminarily assess Cas9 deposition and somatic leakiness across all integrations, we performed genetic crosses between each *vasa1*:Cas9 and *spo11*:Cas9 strain with the gRNA^{dsx} strain and analysed their progeny for the presence of sexual mosaicism. Sexual mosaicism in progeny inheriting only the gRNA but exposed to Cas9 maternally or paternally indicates Cas9 deposition, whereas mosaicism in progeny inheriting both Cas9 and gRNA reflects somatic leakiness. Across all group analyses, no sexual mosaicism was observed when assessing deposition, indicating the absence of Cas9 deposition (Supplementary Table S2).

Regarding somatic leakiness, no sexual mosaicism was observed in *spo11*:Cas9/gRNA^{dsx} individuals. In contrast, a small proportion of trans-heterozygous *vasa1*:Cas9/gRNA^{dsx} individuals exhibited sexual mosaicism at approximately 1% across the three integrations (Supplementary Table S2). As no significant differences were observed among the *vasa1* or *spo11* integrations, we selected the best-

performing strain from each group—based on homing rates and sex distortion efficiency—for more detailed Cas9 deposition analyses.

Specifically, the genetic crosses described above were repeated, and amplicon sequencing at the target site, along with phenotypic assays, were performed on progeny inheriting only the gRNA^{dsx} (Supplementary Figs. S9 and S10). None of the individuals analysed showed sexual mosaicism, mutagenesis at the target site (<0.3% of reads mutated), or fitness costs, suggesting the absence of Cas9 deposition for both the *spo11* and *vasa1* promoters.

To validate these deposition assays, we included data from a previously developed *vasa2*:Cas9 strain. *vasa2*:Cas9 females were crossed to gRNA^{dsx} males, and pooled amplicon sequencing of the gRNA-only progeny showed >99% of reads were mutated, consistent with the strong deposition associated with this promoter (Hammond *et al*., 2016, 2017, 2021). These data have been included in the Supplementary Information (L145–186) and Supplementary Fig. S8, and the raw amplicon sequencing data will be made available.

A similar analysis of the SDMD strain also indicated the absence of Cas9 deposition (Supplementary Fig. S7b).

14. Although this may be outside the scope of this article, bioinformatic analyses of the promoter regions could identify regulatory elements / TF binding sites. This could help in selecting key regions and even allow a reduction in the length of the promoter elements, thereby decreasing the size of the genetic constructs.

We thank the reviewer for this valuable suggestion and fully agree that bioinformatic analysis of promoter regions could provide helpful insights into regulatory elements and transcription factor binding sites, thereby informing future promoter optimisation and construct minimisation. However, a comprehensive bioinformatic and functional characterisation of these promoter regions is beyond the scope of the present study. We plan to pursue this as part of future work aimed at refining and improving the features of the current SDMD strain.

15. Regarding the fitness costs of the initial transgenic lines, the authors have only performed fertility assays, examining hatching rates and larval counts. If data on pupation rates, pupal eclosion or emergence are available, including these would help to understand any other fitness costs associated with these lines.

We thank the reviewer for this helpful suggestion. Survival from the larval to adult stage was monitored in individual progenies of wild-type females crossed to SDMD (n = 24) and wild-type (n = 13) males (Fig. 4i), revealing no significant differences in mortality between the SDMD strain and wild-type controls during the aquatic developmental stages. As additionally suggested by Reviewer #1, we further assessed the fitness of the SDMD strain through a mating competitiveness assay (Fig. 4j). We monitored adult survival for 30 days following pupal eclosion, comparing mortality between SDMD and wild-type individuals. Although a slight, non-significant reduction in mating competitiveness was observed for SDMD males, no differences in adult mortality were detected between SDMD and wild-type individuals. These data have now been included in the revised manuscript (Figs. 4j–k) (L373-387).

16. Also, if the authors have records of the injection experiments performed to develop different Cas9 and SDMD lines, sharing this information would benefit others aiming to develop similar systems.

Records of the injection experiments performed to generate the different Cas9 and SDMD lines have now been provided in Supplementary Table S7.

Minor Comments:

17. Throughout the article, the use of British or American spelling (characterise/characterize, characterised/characterized) was used, and the authors can be consistent.

We thank the reviewer for the comment. All spelling has now been standardised to British English.

18. Microscopy images in Figures 3F & 4D were quite small. Enlarging these would improve clarity. For better utilisation of space, Figures 3D/3G could be combined like Figure 3A.

We thank the reviewer for the suggestion. Figures 3F and 4D have been enlarged, and Figures 3D and 3G have been combined.

19. Line 658/659: “DAPI staining of reproductive organs, 3-day-old mosquito testes or 2-day-old unfed mosquito ovaries were dissected”, I think the data is missing here, or no data is shown.

We thank the reviewer. As figures of testes were not included, the sentence has been adjusted accordingly.

20. Line 196: “<” instead of “£”?

We thank the reviewer for identifying the typo, which has now been corrected.

21. Also, in the figure captions for Figures 2 and 3, “£” symbols were used for the p-values. I think that’s a typo.

We thank the reviewer for identifying the typo, which has now been corrected.

22. Regarding data availability, the authors could also consider providing plasmid sequences. Only the primers used for cloning were listed in the supplementary file – either through a repository or as a GenBank file or similar.

We thank the reviewer for this helpful suggestion. The plasmid sequences will be provided and made publicly available through a suitable repository during the submission process.

23. PS: I really like the Figure 2B schematics of different insertion sites in one figure. Well done!

Reviewer #2 (Remarks on code availability):

Yes, I did check the github code using the above link. The code has a README file and additional information regarding the different packages and dependencies required to run the code. I didn't tried installing / running the code.

Reviewer #3 (Remarks to the Author):

I co-reviewed this manuscript with one of the reviewers who provided the listed reports. This is part of the Nature Communications initiative to facilitate training in peer review and to provide appropriate recognition for Early Career Researchers who co-review manuscripts.

Reviewer #3 (Remarks on code availability):

The code appears to be well structured, but I was unable to test its reproducibility.

It is unclear from this comment whether the reviewer did not try to test reproducibility, or tried and failed. Regardless, we have added an additional file that makes things even easier to run by including all required aspects of the environment in a Jupyter file.

REVIEWER COMMENTS

Reviewer #1 (Remarks to the Author):

I would like to thank the authors for their replies.

However, I am afraid I did not find in the manuscript the text corresponding to these replies in the rebuttal letter:
- “As discussed in the manuscript, we aim to optimise it to further mitigate the potential emergence of functional resistance, for instance by incorporating additional gRNAs. For these reasons, we believe that the extensive and time-consuming nature of cage trials looking at fitness effects would be more appropriately reserved for an optimised version of our construct. We have now added a paragraph to the discussion outlining this reasoning (L562-576).”
In the version of the manuscript I received (natcom_629129_1_uploaded_1762980501.pdf), I did not find such a paragraph L562-576, nor elsewhere.

We apologise for the confusion caused by incorrect line numbering in the submitted document. During preparation of the manuscript for submission, a formatting error occurred in which a block of line numbers was inadvertently omitted, resulting in a misalignment between the line numbers referenced in our responses and those displayed in the manuscript. The relevant revisions and responses can be found in the discussion section as highlighted below:

-Extracted from Discussion-

“Importantly, SDMD systems are predicted to exhibit greater resilience to the emergence and spread of target site resistance compared to SDGD designs, due to the sterility imposed on female carriers (Figs. 1, 6). As functional resistant alleles are positively selected in females, but not in males, ensuring the sterility of the female escapees significantly reduces the effectiveness of selection for resistance, leading to a much prolonged period of time over which the population is suppressed.

No functional resistance has been detected in any previous cage trials of dsx-targeting gene drives, whether or not a sex-distorter element was included, across both small- and large-scale experiments[9, 11, 12, 24, 43]. These results suggest that further cage trials at this locus are unlikely to yield new insights into resistance evolution under laboratory conditions, where population sizes are inherently constrained. Nonetheless, the absence of detectable resistance in laboratory settings does not preclude its emergence following field release, highlighting the importance of the improvements achieved with the SDMD design. To more accurately anticipate the dynamics of resistance evolution, approaches extending beyond conventional cage experiments will be required, with mathematical modelling playing a central role in this effort. In addition, such modelling could incorporate

local, ecological and epidemiological factors[44] to evaluate the precise impact of quantitative reductions in the number of biting females on the burden of malaria.

By employing a CRISPR-Cas9-based X-shredding approach instead of the I-PpoI endonuclease, our design encompasses greater versatility than the previously developed SDGD. This allows for the potential to combine homing and X-shredding in a wider range of insect species[15,16] and to target polymorphisms unique to genetically distinct populations[46].

The SDMD system presented here serves as a proof-of-principle for combining homing and X-shredding via a single promoter and nuclease, simplifying previous SDGD designs and advancing resistance management. However, further optimisation of this construct is needed to enhance its efficacy.

For example, the analysis of the progeny that did not inherit the SDMD construct revealed that about 95% of the alleles were unmutated, suggesting that reduced homing efficiency may result from suboptimal Cas9 activity levels or timing, rather than a preferential use of NHEJ over HDR (Supplementary Fig. S6-S7). Modifying the regulatory elements of vasa1 or incorporating transcriptional enhancers[47] may shed light on whether tuning Cas9 expression can improve homing efficiency without incurring fitness costs in males.

Consistent with our findings and previous studies, homing and sex distortion efficiency are highly locus-dependent[13,37]. Targeting other female fertility genes may optimise both processes, improving the suppression potential of this approach.

In addition, incorporating multiplexed gRNA arrays represents another avenue to reduce the emergence of resistant alleles[9].

To fully evaluate the efficacy of any improved SDMD systems, additional tests on transgenic males, such as flight ability, could be performed to provide a more comprehensive assessment of their fitness.

In summary, the promoters examined in this study provided valuable insights into mosquito gametogenesis and contributed to the advancement of an SDMD technology. While further optimisation of the current design will be advised to evaluate its potential for population suppression fully, the SDMD approach holds considerable promise for the genetic control of the malaria vector, An. gambiae".

- "The observed differences in wild-type larval throughputs likely reflect natural biological variation between the experiments, which were conducted separately. Various factors may have influenced these results, including differences in blood batches and rearing conditions, such as fluctuations in humidity or temperature. For this reason, and to ensure a valid comparison, each experiment included its own wild-type control, treated identically to the experimental group."

I am sorry but strong variations between control groups but also within control groups (from 0 to 120 larvae per female in fig 4f) also reflect poor ability of the team to control heterogeneity (like the quality of the blood…). Using absence of

differences between strains to conclude that fitness are the same is then quite risky. This should be acknowledged as a limitation.

We appreciate the reviewer’s concern and agree that variation in reproductive output can complicate the interpretation of fitness-related traits. Fertility is, however, a complex quantitative trait that is inherently variable and influenced by both genetic and environmental factors across taxa. In Anopheles mosquitoes, substantial variability in larval output among wild-type females, including within the same experimental cohort, has been repeatedly reported and is consistently observed in our laboratory as well as in other laboratories working with this species.

Despite careful standardization of rearing and experimental conditions, factors such as individual physiological status, mating success, blood-meal uptake, and stochastic biological processes contribute to broad distributions in offspring number. Importantly, this degree of variability is not unique to our study and does not necessarily reflect inadequate experimental control, but rather the intrinsic biological heterogeneity of mosquito fertility.

Accordingly, our experimental design follows established practice in mosquito fitness studies by relying on contemporaneous wild-type controls within each experiment and focusing on relative comparisons conducted under identical conditions.

Comparisons between different cohorts were based on arithmetic mean values using the statistical tests reported in the corresponding figure legends. Standard errors are also indicated in the figures, providing an estimate of variability and confidence around the mean.

- “As for the effect of a quantitative reduction (75%) in mosquito population size on malaria incidence, this will depend on the circumstances, but vectorial capacity and the basic reproduction number of the malaria are expected to be linear functions of the number of female mosquitoes. We have now added wording on this point (L572-576).”
Sorry but I could not find this added wording L572-576 or elsewhere in the manuscript. Also, as a specialist of vector-borne disease transmission: no, malaria transmission does not decrease linearly with vector density.

We apologise again for the confusion caused by incorrect line numbering in the submitted document. We appreciate the reviewer’s concern and acknowledge that the wording in this section might lead to confusion.

We did not claim that malaria transmission (which we interpret to mean incidence) decreases linearly with vector density. Rather, we stated that “*at least in the classic Ross-McDonald model, the basic reproduction number is a linear function of the number of biting female mosquitoes*[45].” This statement is demonstrably true, as a cursory look at the cited reference or indeed any review of the Ross-McDonald model will demonstrate. However, it seems this statement (which requires

distinguishing incidence and the basic reproductive number) was confusing, and so we have deleted it, just leaving the statement that the impact of a reduction in the number of biting females on the burden of malaria will depend on various ecological and epidemiological factors, with reference to a recent modelling paper on this topic (Hancock et al. 2024).

This section as now be revised as follow:

"*To more accurately anticipate the dynamics of resistance evolution, approaches extending beyond conventional cage experiments will be required, with mathematical modelling playing a central role in this effort. In addition, such modelling could incorporate local, ecological and epidemiological factors*[44] *to evaluate the precise impact of quantitative reductions in the number of biting females on the burden of malaria*".

Finally, I don't see the point in publishing improved gene drive systems without clearly linking the results to the expected outputs, ie vector suppression else than from model predictions and more importantly malaria incidence. Here you show a better resilience to resistance, but not efficiency, contrary to what is stated in the title.

We acknowledge the reviewer's comment regarding the scope of the conclusions and the wording of the title. In response, we have revised the title from "*Sex Distorter Male Drive for efficient, resistance-resilient population control of the human malaria vector Anopheles gambiae*" to "*Sex Distorter Male Drive for resistance-resilient population control of the human malaria vector Anopheles gambiae*". This change better reflects the primary focus of the manuscript, which is the enhanced resilience to resistance rather than demonstrated improvements in population suppression efficiency or downstream epidemiological outcomes.

Reviewer #2 (Remarks to the Author):

The authors have addressed all my comments and revised the manuscript. I am satisfied with the changes, and I believe the quality has improved.

We sincerely thank the reviewer for their time and constructive feedback.

Reviewer #3 (Remarks to the Author):

I co-reviewed this manuscript with one of the reviewers who provided the listed reports. This is part of the Nature Communications initiative to facilitate training in peer review and to provide appropriate recognition for Early Career Researchers

who co-review manuscripts.

Reviewer #3 (Remarks on code availability):

Yes, the code is reproducible, usable, and includes clear instructions for installation and execution.

We sincerely thank the reviewer for their time and constructive feedback.
